# Astrocytes derived from ASD individuals alter behavior and destabilize neuronal activity through aberrant Ca^2+^ signaling

**DOI:** 10.1038/s41380-022-01486-x

**Published:** 2022-04-01

**Authors:** Megan Allen, Ben S. Huang, Michael J. Notaras, Aiman Lodhi, Estibaliz Barrio-Alonso, Pablo J. Lituma, Paul Wolujewicz, Jonathan Witztum, Francesco Longo, Maoshan Chen, David W. Greening, Eric Klann, M. Elizabeth Ross, Conor Liston, Dilek Colak

**Affiliations:** 1 Center for Neurogenetics, Feil Family Brain and Mind Research Institute, Weill Cornell Medicine, Cornell University, New York, NY USA; 2 Department of Psychiatry, Weill Cornell Medicine, Cornell University, New York, NY USA; 3 Center for Neural Science, New York University, New York, NY USA; 4 Molecular Proteomics, Baker Heart and Diabetes Institute, Melbourne, VIC Australia; 5 Baker Department of Cardiometabolic Health, The University of Melbourne, Melbourne, VIC Australia; 6 Baker Department of Cardiovascular Research, Translation and Implementation, La Trobe University, Melbourne, VIC Australia; 7 Central Clinical School, Monash University, Melbourne, VIC Australia; 8 Gale and Ira Drukier Institute for Children’s Health, Weill Cornell Medicine, Cornell University, New York, NY USA

**Keywords:** Neuroscience, Diseases

## Abstract

The cellular mechanisms of autism spectrum disorder (ASD) are poorly understood. Cumulative evidence suggests that abnormal synapse function underlies many features of this disease. Astrocytes regulate several key neuronal processes, including the formation of synapses and the modulation of synaptic plasticity. Astrocyte abnormalities have also been identified in the postmortem brain tissue of ASD individuals. However, it remains unclear whether astrocyte pathology plays a mechanistic role in ASD, as opposed to a compensatory response. To address this, we combined stem cell culturing with transplantation techniques to determine disease-specific properties inherent to ASD astrocytes. We demonstrate that ASD astrocytes induce repetitive behavior as well as impair memory and long-term potentiation when transplanted into the healthy mouse brain. These in vivo phenotypes were accompanied by reduced neuronal network activity and spine density caused by ASD astrocytes in hippocampal neurons in vitro. Transplanted ASD astrocytes also exhibit exaggerated Ca^2+^ fluctuations in chimeric brains. Genetic modulation of evoked Ca^2+^ responses in ASD astrocytes modulates behavior and neuronal activity deficits. Thus, this study determines that astrocytes derived from ASD iPSCs are sufficient to induce repetitive behavior as well as cognitive deficit, suggesting a previously unrecognized primary role for astrocytes in ASD.

## Introduction

Autism spectrum disorder (ASD) is a developmental disability characterized by impaired social communication, restrictive repetitive behaviors, and cognitive deficits. Nearly 95% of ASD diagnoses are not associated with a known genetic mutation(s) [1]. Our lack of knowledge about the cell types that participate in disease progression early in brain development has limited the generation of effective therapies. While ASD is highly heritable (40–80%), uncovering common genetic biomarkers through sequencing and linkage studies remains challenging [2–4]. This suggests that a multitude of rare genetic variants converge on limited biological pathways to cause ASD. Recent work has begun to cluster these variants and indicate that most play a role in neural communication and plasticity [2, 5, 6].

The onset of ASD symptoms coincides with activity-dependent synaptic plasticity events [7]. In typically developing children, intense neuroplasticity occurs in the first few years of life, a time which parallels significant astrocyte proliferation and refinement [8]. In fact, astrocytes are in close physical contact with neuronal cell bodies, dendrites, and dendritic spines. A single mouse cortical astrocyte contacts over 100,000 synapses, whereas a human astrocyte contacts up to 2,000,000 synapses [9–11]. This intimate physical relationship supports the instructive roles astrocytes play in several key plasticity processes, including the formation of synapses [12–14], secretion of factors that affect enhance structural changes like spine formation and dendritic arborization [15–18], pruning of supernumerary synapses [19], and the removal of excess neurotransmitters to prevent excitotoxicity [20]. Further, astrocytes have been implicated in the maintenance of learning and memory through the modulation of long-term potentiation (LTP) [21]. LTP, one of the major forms of synaptic plasticity, represents the cellular basis for learning and memory [22]. However, whether human astrocyte pathology plays a causal role in nonsydromic ASD, as opposed to a compensatory response to an already diseased brain, is not clear.

Astrocytes have been implicated in the pathogenesis of mouse models of syndromic ASDs. Co-culture experiments revealed that the presence of astrocytes derived from the brains of two syndromic mouse models, Rett and fragile X syndromes, negatively altered neuronal structure [23, 24]. Additionally, transcriptomic and immunohistochemistry studies in postmortem tissues found enrichment of astrocytic reactivity in the cortex of brains from individuals with ASD [25–27]. ASD iPSC-derived astrocytes caused a decrease in neurite number and synaptic markers in iPSC-derived neurons, suggesting a potential contribution of astrocyte dysfunction in ASD pathophysiology [28]. However, specific behavioral alterations as well as mechanisms through which astrocytes contribute to ASD remain unknown.

Here, we show that the presence of ASD astrocytes in a healthy brain induces repetitive behavior as well as memory and synaptic plasticity deficits. Three independent experiments, unbiased proteomic analysis, in vitro two-photon live-cell imaging, and live-animal two-photon imaging, commonly identified that altered Ca^2+^ signaling is an inherent defect in astrocytes derived from multiple individuals with ASD. Stem cell culturing methods were combined with transplantation to generate chimeras to study how astrocytes derived from ASD iPSCs function in vivo. Transplanted ASD astrocytes exhibit elevated Ca^2+^ responses. We report that ASD astrocytes induce repetitive behavior as well as memory impairments, which are accompanied by reduced LTP in hippocampal slice cultures from ASD chimeras. In line with these phenotypes, ASD astrocytes reduce spine density and neuronal network activity when co-cultured with wild type (WT) hippocampal neurons in vitro. Modulation of evoked Ca^2+^ release in ASD astrocytes protects against deficits in neuronal network dynamics and fear memory behavior. In summary, these data define a mechanistic role for astrocytes in ASD and provide insight into our understanding of ASD pathogenesis.

## Aberrant Ca^2+^ activity in ASD astrocytes

To study astrocyte pathology in ASD, we used iPSCs derived from control (CTRL) and ASD individuals. In total, nine distinct CTRL (7 male, 2 female) and nine distinct ASD (all male) iPSC lines were used. Please refer to Supplementary Table 1 for details about clinical information, ADOS scores, age of sampling, race, etc. as well as identifier number for purchasing information. Whole exome sequencing was performed in astrocytes derived from all CTRL and ASD iPSC lines used in this study (Supplementary Tables 2 and 3).

**Fig. 1:**
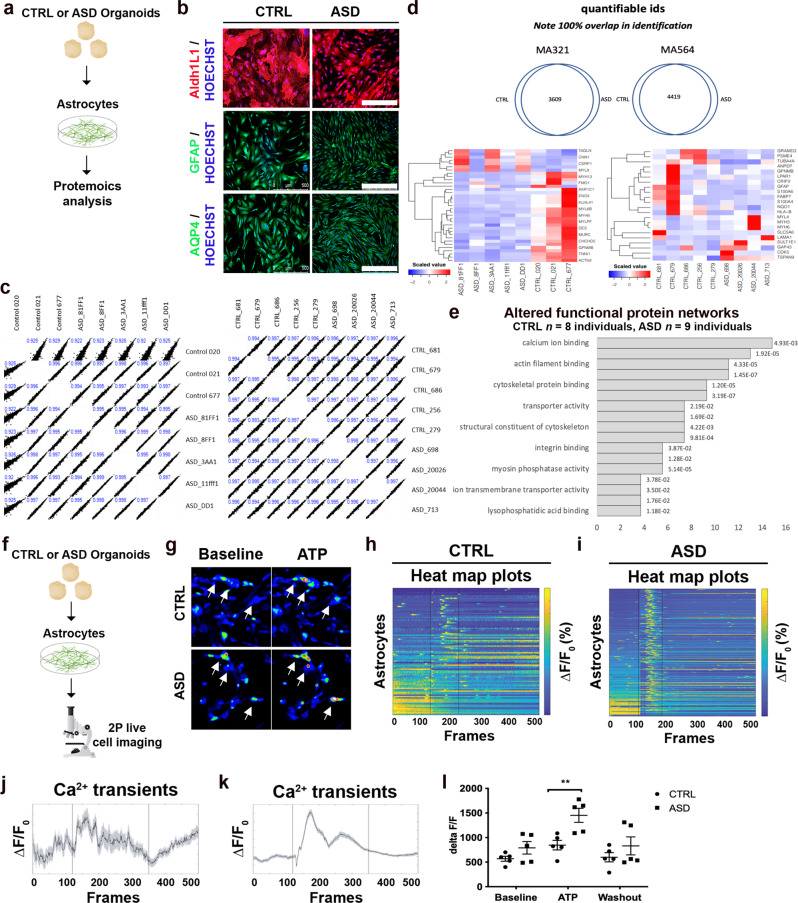
Aberrant Ca^2+^ activity in ASD astrocytes. **a**, **b** Spontaneous generation of human astrocytes. **a** Astrocytes were dissociated from ASD or CTRL organoids at day 75 and expanded in culture (see “Methods” section). **b** Representative images from immunostainings shows that astrocytes dissociated from organoids expressed multiple astrocyte markers: ALDH1L1, GFAP, and AQP4 as well as Vimentin and S100Beta (see also Supplementary Fig. 1b–d). **c**–**e** Proteomic study identified Ca^2+^ signaling as the most significantly altered network in ASD astrocytes. Proteins were extracted from astrocytes and labeled with Tandem Mass Tag (TMT) chemistry followed by LC/MS analysis. **c** Two independent proteomic runs and analyses, of which each exhibited high experimental reproducibility. **d** Venn diagrams for both runs revealed 3609 and 4419 proteins, respectively, which were common to both CTRL and ASD astrocyte samples. **e** GO analysis revealed enrichment for Ca^2+^ ion binding proteins in ASD samples. **f**–**i** Increased Ca^2+^ activity in ASD astrocytes. **f** CTRL or ASD astrocytes were loaded with Ca^2+^ indicator dye (Fluo-4-am, 1 μM). Data are shown as the change in fluorescent activity divided by baseline fluorescent activity (Δ*F*/*F*_0_). **g** Representative still images taken from the imaging videos highlighted fluorescent activity under baseline conditions (left) and after application of 50 μM ATP (right). Arrows point to cells that expressed Ca^2+^ transients under baseline conditions (left) and after stimulation with ATP (50 μM). ASD astrocytes responded to stimulation with more intense transients as evidenced by increased fluorescence (hotter color) (see also Supplementary Video 1). Representative heat maps of Ca^2+^ responses from CTRL (**h**) or ASD (**i**) astrocytes across time visually confirmed enhanced evoked responses from ASD astrocytes (application of ATP occurred within black vertical lines). Evoked responses (Δ*F*/*F*_0_) from all CTRL (**j**) and ASD (**k**) recordings, sampled over multiple days of recording and from multiple ASD lines, were plotted as a function of time (frames). **l** Quantification of the maximal peak amplitude of Ca^2+^ upon application of ATP showed that, when compared to CTRL astrocytes, ASD astrocytes exhibited increased Ca^2+^ activity in response to ATP. In summary, two independent experiments confirmed that ASD astrocytes harbor dysfunctional Ca^2+^ signaling. Scale bar = 500 μm. Data are represented as mean ± SEM. Proteomics analysis: CTRL *n* = 8 lines; ASD *n* = 9 lines. Two-photon Ca^2+^ imaging: CTRL *n* = 865 cells from five lines; ASD *n* = 847 cells from five lines.

We adapted an undirected-differentiation organoid system [29, 30] to isolate astrocytes derived from CTRL or ASD lines (Fig. 1a; see also Supplementary Fig. 1a). Undirected organoid protocols recapitulate the temporal sequence of cortical development seen in developing embryos [31], and so, organoids derived from ASD iPSCs spontaneously generate astrocytes in an environment that mimics the early ASD brain. This is important because astrocytes require early interaction with neurons to induce expression of critical receptors and trigger temporally regulated activation patterns [32]. Organoids were enzymatically dissociated at 75 days in vitro (DIV), and astrocyte enrichment was selected with culture medium supplemented with glucose and low serum (2%) as previously described [33]. While serum might select for specific astrocytic phenotypes, it is required for astrocyte expansion. Astrocytes grown in the selection medium expressed numerous astrocyte markers including ALDH1L1, GFAP, and AQP4 (Fig. 1b) as well as Vimentin and S100Beta (Supplementary Fig. 1b, c) by passage 8. Additionally, CTRL and ASD astrocytes were not reactive in culture conditions (Supplementary Fig. 2).

We first took an unbiased approach and compared the protein profiles of 9 ASD astrocytes to 8 CTRL astrocytes with Tandem mass tag (TMT) liquid chromatography mass spectrometry (Fig. 1c–e) [34]. Notably, proteomic analysis validated the method of astrocyte extraction as several markers for astrocyte identity were detected (Supplementary Fig. 3) [35, 36]. Proteomics analysis was split into two label-based protein quantification analyses using TMT chemistry. This approach elicited a median depth of 4014 protein quantifications. Quantifiable values were also highly reproducible between samples and runs (Fig. 1c). This demonstrates high experimental reproducibility and cell-type homogeneity. For each sample group, differentially regulated proteins constituted 22 (0.969% of Run 1) and 35 proteins (0.497% of Run 2) of each analysis group, respectively (Fig. 1d). Higher magnification of heat maps and individual gene names are presented in Supplementary Fig. 3e, f, respectively.

Coverage and enrichment in Molecular Function (MF) GO categories revealed Ca^2+^ ion binding as the most enriched MF category (Fig. 1e). Qiagen’s ingenuity pathway analysis (IPA) once more determined Ca^2+^ signaling as the most significantly altered pathway. To functionally confirm this predicted deficit, Ca^2+^ activity was assessed in cultured astrocytes isolated from CTRL and ASD organoids using two-photon live-cell imaging (Fig. 1g–i). CTRL and ASD heat map plots demonstrated the exaggerated response to ATP (50 μM) application (Fig. 1h, i and Supplementary Video 1). Evoked responses (Δ*F*/*F*_0_) [37] from all recordings, taken over multiple days of recording and from multiple lines, were plotted as a function of time (frames) (Fig. 1j, k). Maximal peak amplitude of Ca^2+^ showed that, when compared to CTRL astrocytes, ASD astrocytes exhibited increased Ca^2+^ activity in response to ATP (Fig. 1l).

Taken together, these data suggest that ASD astrocytes respond to stimulation with increased Ca^2+^ responses.

## Organoid-derived astrocytes migrate throughout the mouse cortex and survive into adulthood

**Fig. 2:**
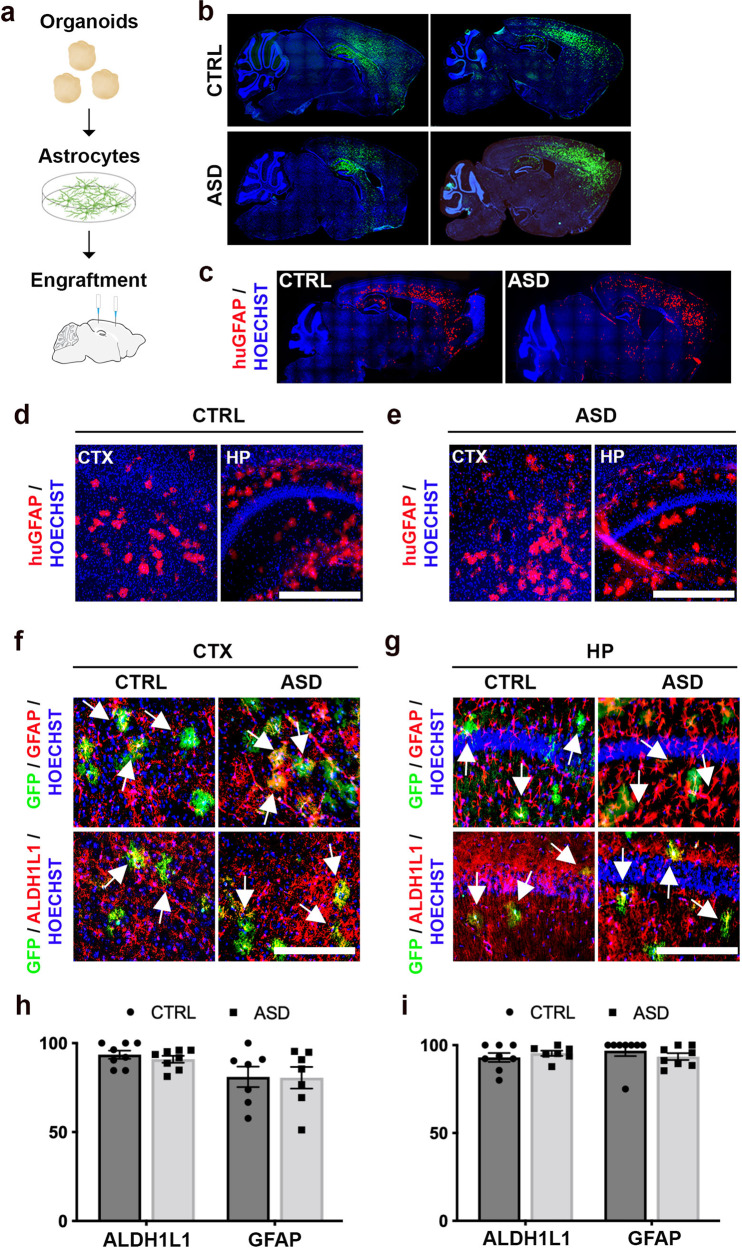
Organoid-derived astrocytes migrate throughout the mouse cortex and survive into adulthood. **a** Schematic of experimental workflow. We transplanted a total of 8–10 × 10^5^ astrocytes (infected with CAG-GFP virus) into the brains of *Rag2*^*KO*^ neonatal mice, spread out over four injection sites, which were bilateral along the midline, anterior and posterior to bregma. **b**–**e** Immunostained whole brain slices cut on the sagittal plane both for GFP (**b**) and the human-specific GFAP epitope (huGFAP) at P60 showed a wide and homogenous spread throughout the cortex (**c**) (see also Supplementary Fig. 4 for stereological quantifications). **d**, **e** Representative higher magnification images illustrated the spread of huGFAP in cortex and hippocampus of chimeric mice. **f**–**i** Representative co-immunostained images of chimeric brains revealed high co-localization of GFP expression (human astrocytes, green) with the two astrocyte markers, GFAP (red, top panel) and ALDH1L1 (red, bottom) in the cortex (**f**) and hippocampus (**g**). Arrows indicate examples of dual positive cells. More than 90% of GFP+ cells expressed astrocyte markers suggesting that astrocytes retained their identities upon maturation in the adult mouse brain (**h** and **i**, see also Supplementary Fig. 5). These results establish that CTRL and ASD astrocytes dissociated from organoids generated homogenous transplantations in host brains. CTX Cortex, HP Hippocampus. Scale bar = 500 μm for **d** and **e** and 250 μm for **f** and **g**. Data are represented as mean ± SEM. Co-localization: CTRL and ASD *n* = 8 per group (2 mice/2 distinct lines, and 4 slices per brain).

To determine whether ASD astrocytes induce behavioral deficits, GFP-expressing CTRL or ASD astrocytes were transplanted into the brains of neonatal (postnatal days 1–3 [P1-3]) mice using an established multisite-injection protocol with minor modifications [38]. *Rag2*^*KO*^ immune-compromised mice was used as transplant hosts to limit rejection of human astrocytes [38–40]. GFP immunostainings in whole brain sagittal sections highlighted extensive migration throughout the cortex at P60 (Fig. 2b). In addition to GFP labeling, astrocyte survival and migration in chimeric brains was also confirmed by immunostaining against a human-specific GFAP epitope [41] (Fig. 2c–e). To quantify the number of human astrocytes that survived in the chimeric brains, stereological cell counting using the optical fractionator method was performed. No significant difference in the number of surviving human astrocytes in ASD astrocyte chimeric brains relative to CTRL astrocyte chimeric brains was found (Supplementary Fig. 4). Similarly, engrafted cells were positioned both in anterior-posterior and medio-lateral axes in cortex in both groups (Supplementary Fig. 4). Chimeric brains were co-immunostained for GFP and either GFAP or ALDH1L (Fig. 2f, g). More than 90% of human GFP+ cells co-expressed astrocyte markers in adult chimeric brains (Fig. 2h, i). Examination of over 800 GFP+ cells in both groups showed that none of the GFP+ cells expressed neuron marker NeuN (Supplementary Fig. 5).

## In vivo imaging confirms aberrant Ca^2+^ activity in ASD astrocytes

**Fig. 3:**
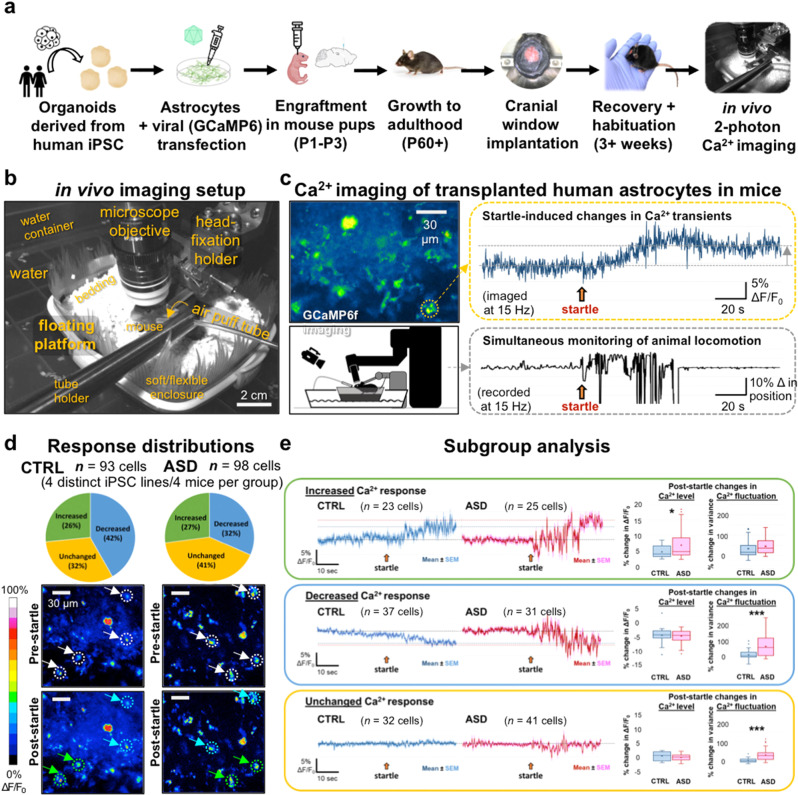
In vivo imaging confirms aberrant Ca^2+^ activity in ASD astrocytes. **a** Schematic of experimental workflow. **b** In vivo imaging setup. Photograph with labeled components of our custom-designed floating platform, developed to provide a tactile virtual-reality environment for head-fixed mice and to enable imaging of actively locomoting animals with minimal motion confounds (see “Methods” for details). **c** Ca^2+^ imaging of transplanted human astrocytes in mice. Left, upper panel: a representative image of GCaMP6f-expressing human astrocytes in the cortex of a mouse engrafted with cells derived from a CTRL human subject. Right, upper panel (orange box): Ca^2+^ transient recorded from the cell marked by the orange circle on the left image, showing an increase in Ca^2+^ level after the startle/air-puff stimulus (orange arrow). Left bottom panel: schematic of the imaging setup. The mouse is situated at the center on top of an enclosed platform (boat) floating on water (see “Methods” for details). Right bottom panel (gray box): representative trace of recorded locomotion during imaging, produced by analyzing the infrared video recording and plotting the light intensity change of a select ROI (region of interest) on the floating platform. As animal movement directly translated into platform displacement, any body motion generated by the animal could be tracked with accuracy and high temporal resolution, even heavy breathing could be seen indicated by the brief ticks at the latter part of the recording. **d** Response distributions. Transplanted human astrocytes displayed a variety of Ca^2+^ activity responses in vivo in reaction to the air-puff startle stimulus, including increased, decreased, and unchanged Ca^2+^ levels. We segmented the cells with a 20-μm-diameter mask, which encompassed both soma and processes. Top: pie charts showing the percent distribution of each response types in CTRL and ASD astrocytes. Bottom: representative images of CTRL and ASD astrocytes before and after startle. White circles and arrows (upper panels) point to sample cells showing post-startle increased (green arrows/circles) and decreased (blue arrows/circles) Ca^2+^ responses. **e** Subgroup analysis. For all traces, the mean ± SEM are plotted, representing the population average. Top (green box): increased response (cells displaying a positive change in Δ*F*/*F* after startle). Box plots showing ASD cells exhibit a significantly larger increase than CTRL cells (post-startle % change in Δ*F*/*F*: CTRL: +4.66 ± 0.384%, *n* = 23; ASD: +6.81 ± 0.937%, *n* = 25; unpaired *t*-test, *p* = 0.04). No significant differences are found in the changes in fluctuation between CTRL and ASD cells. Middle (blue box): decreased Ca^2+^ response subgroup (cells showing negative change in Δ*F*/*F* after startle). Both CTRL and ASD show decreases in Ca^2+^ to similar levels (~ −5%). However, there is a drastic difference in their change in Ca^2+^ fluctuations. Compare the flat downward sloping blue trace vs. the red trace with the dramatic fluctuation after startle. The box plots quantitatively illustrate this difference (Post-startle change in Ca^2+^ fluctuation: CTRL: 7.08 ± 4.74%, *n* = 37; ASD: 60.74 ± 11.04%, *n* = 31; unpaired *t*-test, *p* = 0.000062). Bottom (orange box): unchanged Ca^2+^ response subgroup (cells whose changes in Ca^2+^ levels are between +2 and −2%). While CTRL cells in this group show a completely flat and unresponsive activity profile, the ASD cells with unchanged Ca^2+^ levels showed a highly significant change in Ca^2+^ fluctuation (30% increase in variance) (Post-startle change in Ca^2+^ fluctuation: CTRL: 1.39 ± 2.08%, *n* = 32; ASD: 30.75 ± 4.63%, *n* = 41, unpaired *t*-test, *p* = 0.00000036).

Astrocyte Ca^2+^ transients are best captured in vivo where astrocytes display their mature and complex morphology [42]. To measure Ca^2+^ responses in human astrocyte chimeric mice, we used genetically encoded indicators and cranial window implantation followed by two-photon imaging as previously described [43] (Fig. 3a–c). Human astrocytes carrying the genetically encoded calcium indicator GCaMP6f (AAV2/5-*GfaABC*_*1*_*D-GCaMP6f*) were engrafted into neonatal *Rag2*^KO^ (P1-3). At P60+, a 3-mm-diameter glass cranial window was implanted over the primary somatosensory and motor cortices (S1/M1) (see “Methods”). After recovery, Ca^2+^ activity was recorded by two-photon imaging in engrafted human astrocytes (see “Methods”). Transplanted human astrocytes displayed a variety of Ca^2+^ activity responses in vivo in reaction to an air-puff startle stimulus, including increased, decreased, and unchanged Ca^2+^ levels (Fig. 3d). When categorized, these response groups revealed significant differences between Ca^2+^ activity in CTRL and ASD astrocytes. In the increased response group, ASD astrocytes exhibited a significantly larger increase than CTRL cells (Fig. 3e, top). While decreases in Ca^2+^ levels were not significant between the groups, ASD astrocytes displayed a drastic increase in Ca^2+^ fluctuations compared to the CTRL astrocytes in decreased Ca^*2+*^ response subgroup (Fig. 3e, middle). Similarly, while CTRL cells showed a completely flat and unresponsive activity profile, the ASD cells with unchanged Ca^2+^ levels displayed a highly significant change in Ca^2+^ fluctuations (Fig. 3e, bottom).

Together, in vivo Ca^2+^ imaging of transplanted human astrocytes in awake behaving mice indicate that ASD astrocytes exhibit significantly heightened responses to environmental stimuli, as seen both in significantly increased Ca^2+^ elevation as compared to CTRL astrocytes and highly significant increases in all ASD astrocytes in response to the startle stimulus despite decrease or unchanged Ca^2+^ levels.

## ASD astrocyte chimeric mice exhibit repetitive behavior as well as impaired memory and hippocampal LTP

We next sought to assess whether ASD astrocyte chimeric mice display cognitive and behavioral abnormalities. In addition to sociability and perseverative behavior, memory deficits and learning difficulties are found throughout the autism spectrum [44–50]. In fact, the ASD iPSC lines used in this study were derived from ASD individuals who scored low in IQ tests (Supplementary Table 1). Fear-conditioning paradigms are explicitly recommended to investigate the breadth of emotional impairment in affected individuals [51, 52]. Thus, adult CTRL and ASD chimeric mice were subjected to a range of ethologically relevant behavioral assays. ASD chimeric mice did not demonstrate differences in exploratory or general activity relative to CTRL chimeric mice (Supplementary Fig. 6), suggesting that transplantation did not affect ambulatory activity and is not a confounding factor in the interpretation of other behavioral results. Anxiety-like behavior was also assessed by measuring time spent in the center of the photocells in the Open Field. No evidence of anxiety-like behavior between CTRL and ASD chimeric mice emerged from this test, suggesting that ASD astrocytes did not overtly induce anxiety-like behavior (Supplementary Fig. 6).

**Fig. 4:**
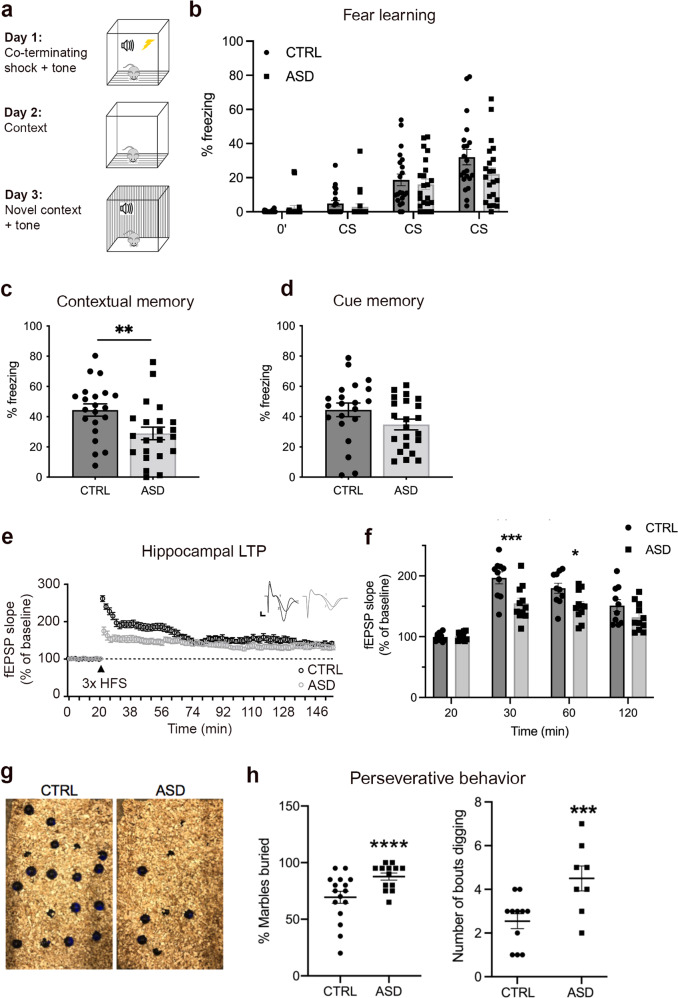
ASD astrocyte chimeric mice exhibit impaired fear memory and hippocampal LTP along with repetitive behavior. **a**–**d** ASD astrocyte chimeric mice displayed deficits in fear memory but not in fear learning. **a** Schematic summarizing classical fear-conditioning paradigm. On day 1, mice were trained to associate an audible tone (30 s duration, 70 dB) with a co-terminating foot shock (1-s duration, 0.7 mA). Testing days 2 and 3 measured freezing behavior in response to exposure to the training context or an audible cue in a novel context, respectively. Freezing behavior in the testing trials provided a quantifiable measure of fear memory (see also “Methods”). **b** There was no significant difference in the rate of acquisition learning between CTRL and ASD mice (ANOVA with Bonferroni posthoc test *p* value >0.05). **c** ASD chimeric mice showed reduced freezing behavior when exposed to the fear context (unpaired *t*-test = 0.009). **d** No differences were found in the freezing behavior between CTRL or ASD chimeric mice during cue presentation in a novel context (unpaired *t-*test = 0.10). **e**, **f** LTP was tested as a synaptic correlate of learning and memory in hippocampal brain slices (400 µm) of 4–6-month old transplanted mice. ASD astrocyte chimeric brain slices showed reduced potentiation in the early phase of LTP compared to control (30–60 min). ASD astrocyte chimeric brain slices displayed the greatest differences in the fEPSP slope within the first 60 min of recording (**f**). **g**, **h** To test ASD chimeric mice for repetitive behavior, we employed the marble burying test as well as monitored circling and backflipping. Marbles were scored as buried if at least 60% of the marble was covered (30-min period) (**g**). ASD astrocyte chimeric mice buried significantly more marbles when compared to CTRL astrocyte chimeric mice (unpaired *t-*test = 0.009) but exhibited no circling or backflipping (**h**). Taken together, these results indicate that ASD astrocytes induce ASD-related perseverative behavior, memory dysfunction, and hippocampal LTP deficits. Data are represented as mean ± SEM. Fear testing: CTRL *n* = 21 male and female mice/4 distinct lines, ASD *n* = 22 male and female mice/3 distinct lines; LTP: CTRL *n* = 10 slices, 4 mice/2 distinct lines; ASD *n* = 10 slices, 5 mice/2 distinct lines). Marble burying behavior: CTRL *n* = 16 mice/5 distinct lines, ASD *n* = 13 mice/5 distinct lines.

To assess learning and memory in ASD and CTRL astrocyte chimeric mice, a classical fear-conditioning protocol was used (Fig. 4a). Fear-conditioning is an associative learning and fear memory task where mice are trained to associate a neutral conditioned stimulus (audible tone) with an aversive unconditioned stimulus (mild electrical foot shock) and display a conditioned response (freezing behavior). The freezing behavior is used as an index of the mouse’s ability to learn the task and later recall the fear memory (see “Methods”). ASD astrocyte chimeric mice exhibited impaired contextual memory relative CTRL astrocyte chimeric mice (Fig. 4c, d). Because there is a male bias in ASD prevalence [53], sex split analyses were performed for fear learning and fear memory tests (Supplementary Fig. 7). Two-way ANOVA revealed no significant effect of sex, nor an interaction of sex and ASD transplantation status. The only factor to reach significance was ASD diagnosis. Therefore, sex differences did not contribute to fear memory deficits seen in ASD chimeric mice (Supplementary Fig. 7). Spatial learning and memory were also assessed in ASD astrocyte chimeric mice by Morris water maze (MWZ), where a test mouse relies on distal cues to locate a hidden escape platform submerged in opaque water (see “Methods”). No spatial learning and memory deficits were detected in ASD astrocyte chimeric mice in the MWZ (Supplementary Fig. 8a–d).

Persistent changes in synaptic strength via LTP represent a cellular mechanism for the formation and retention of memories [54]. Intriguingly, altered LTP has been implicated in multiple syndromic models of ASD [55]. Astrocyte support is not only required for proper synaptic plasticity [56–59], but also enhances LTP and memory [60]. LTP is composed of two different phases [61]: protein synthesis-independent early-phase LTP (E-LTP) and protein synthesis-dependent late-phase LTP (L-LTP). ASD astrocyte chimeric slices showed reduced potentiation in the early phase of LTP compared to CTRL astrocyte chimeric slices (Fig. 4e, f).

ASD diagnosis is based on three categories of behavioral criteria: abnormal social interactions, communication deficits and repetitive behaviors [62]. To probe the role ASD astrocytes play in perseverative behaviors, a marble burying test was used [63, 64]. ASD astrocyte chimeric mice buried significantly more marbles compared to CTRL chimeric mice indicating that ASD astrocytes induced a form of repetitive-like behavior (Fig. 4g, h). Similar to the fear memory phenotype, sex split analyses showed that sex did not produce a main effect, nor was an interacting factor that contributed to perseverative digging behavior (Supplementary Fig. 7). To score sociability, a three-chamber social interaction paradigm was used as previously described [15, 65, 66]. In the sociability test, ASD astrocyte chimeric mice spent similar amounts of time in the social and nonsocial zones relative to CTRL astrocyte chimeric mice (Supplementary Fig. 8g).

Results from these experiments indicate that ASD astrocytes can induce repetitive behavior as well as cause selective memory and synaptic plasticity deficits.

## ASD astrocytes decrease neuronal network firing and spine density in vitro

**Fig. 5:**
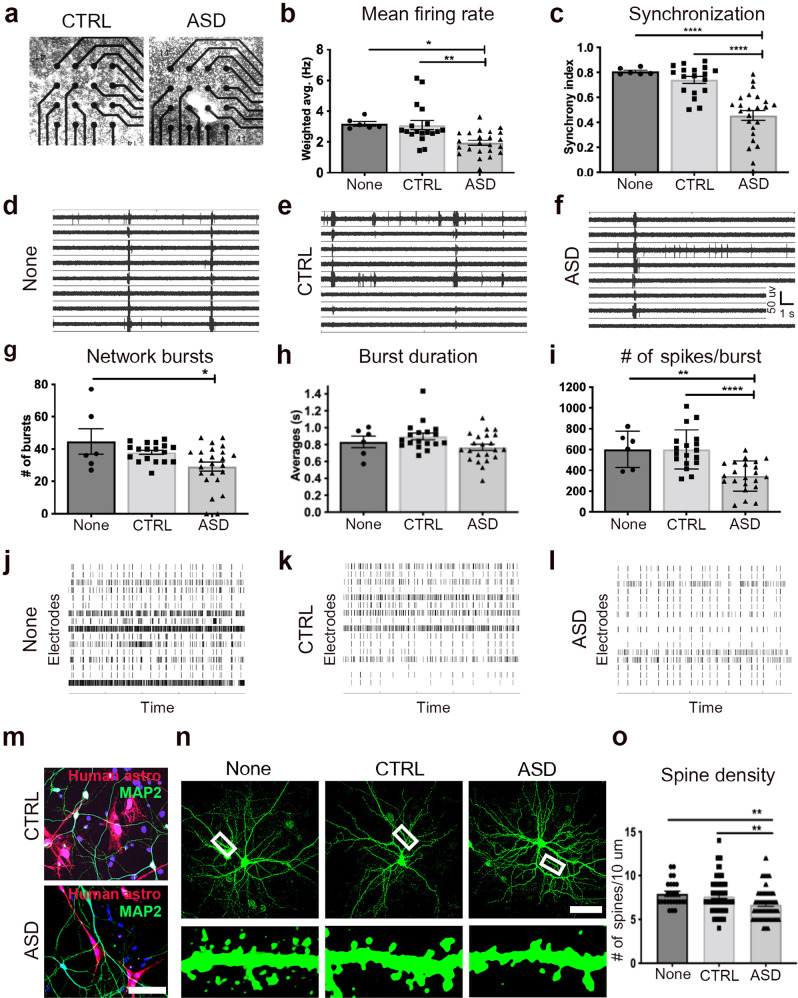
ASD astrocytes decrease neuronal network firing and spine density in vitro. **a**–**l** Primary hippocampal neurons dissociated from E16-18 WT mouse brains were co-cultured with human astrocytes isolated from CTRL or ASD organoids to measure spontaneous network activity with MEA. CTRL cultures refer to co-culturing of CTRL human astrocytes with hippocampal neuronal cultures (naturally containing some mouse astrocytes), and ASD cultures refer to co-culturing of ASD human astrocytes with hippocampal neuronal cultures (naturally containing some mouse astrocytes). “None” cultures refer to hippocampal neuronal cultures (containing some mouse astrocytes) without any addition of human astrocytes. **a** Representative images of co-cultures plated in a single well of a 48-well array plate. **b** ASD astrocyte co-cultures displayed decreased mean network firing rate when compared to CTRL co-cultures and None. (ANOVA with Tukey’s posthoc test None vs. CTRL *p* value = 0.98, None vs. ASD *p* value = 0.02, CTRL vs. ASD *p* value = 0.002, see also Supplementary Video 2). **c** ASD astrocytes also disrupted network synchronization when compared to CTRL astrocytes and None (None vs. CTRL *p* value = 0.65, None vs. ASD *p* value <0.0001, CTRL vs. ASD *p* value < 0.0001). **d**–**f** Representative raw traces of spontaneous spiking activity over a 10-s period. **g** ASD astrocytes decreased the # of network bursts when compared to None (None vs. ASD *p* value = 0.02, CTRL vs. ASD *p* value = 0.06). **h**, **i** ASD astrocytes did not affect average burst duration but decreased the # of spikes per burst network (None vs. ASD *p* value = 0.005, CTRL vs. ASD *p* value <0.0001). **j**–**l** Representative raster plots (4-min) demonstrated the decrease in burst number and spikes per bursts in the ASD co-culture group compared to CTRL co-culture and None. **m**–**o** Spine density quantified in a 10 μm dendritic segment at least 20 μm away from the soma in hippocampal neurons co-cultured with ASD or CTRL human astrocytes. **m** Immunostained co-cultures with human astrocytes infected with CMV GFP lentivirus (pseudo colored red) prior to co-culture. **n** Neurons were labeled with a αCamKII GFP AAV at DIV5 and fixed at DIV18 (10 μm dendritic segments shown at bottom). **o** ASD astrocytes decreased spine density on primary hippocampal neurons (None vs. CTRL *p* value = 0.78, None vs. ASD 0.009, CTRL vs. ASD *p* value = 0.004). These results provide direct evidence that ASD astrocytes influence structural and functional properties of neurons that weaken electrophysiological activity. Scale bar = 100 μm. Data are represented as mean ± SEM. MEA: None *n* = 6 wells, CTRL *n* = 18 wells, 3 distinct lines; ASD *n* = 22–24 wells, 4 distinct lines. Spine quantification: None *n* = 25 neurons, CTRL *n* = 71 neurons co-cultured with 4 distinct lines, ASD *n* = 81 neurons co-cultured with 5 distinct lines. CTRL: Co-cultures of CTRL human astrocytes with mouse hippocampal neurons. ASD: Co-cultures of ASD human astrocytes with mouse hippocampal neurons. None: Mouse hippocampal neuron cultures with no human astrocytes.

To gain mechanistic insight into how ASD astrocytes induce behavioral and LTP deficits in chimeric brains, we simulated the in vivo macroenvironment by co-culturing ASD astrocytes with mouse hippocampal neurons and assessed the effects of ASD astrocytes on neuronal structure and function. Hippocampal neuronal cultures are inherently a mixture of glutamatergic neurons (over 70%) and astrocytes (<30%) (Supplementary Fig. 9). Primary hippocampal cells dissociated from WT embryonic mouse brains at embryonic days 16 to 18 (E16-18) were cultured with human astrocytes isolated from CTRL or ASD organoids. These two co-culture conditions mimic closely the in vivo cellular interactions of our chimeric models. Astrocytes mediate connectivity and synchronized network activity [19, 67, 68]. Thus, At DIV14, spontaneous network activity was measured using multi-electrode array (MEA). Co-cultures were plated on 48-well MEA plates with a 4:1 hippocampal cell to human astrocyte ratio (Fig. 5a). In all co-culture experiments, an additional control culture dissociated from the same litter that consisted of primary hippocampal cells with no human astrocytes (None) were included.

Addition of ASD astrocytes to hippocampal neurons decreased the mean firing rate compared to the neurons co-cultured with CTRL human astrocyte and None (Fig. 5b and Supplementary Video 2). ASD astrocytes also disrupted synchronous firing (Fig. 5c). Representative raw spike plots further illustrated the diminished mean firing and reduced synchronicity in the ASD co-cultures relative to CTRL co-cultures and None (Fig. 5d–f). Network bursts occur when groups of neurons fire coordinated trains of spikes in specified patterns. ASD astrocytes showed a decreased number of network bursts relative to None (Fig. 5g) and decreased number of spikes per burst compared to CTRL co-cultures and None (Fig. 5i). However, ASD astrocytes did not alter burst duration in these experiments (Fig. 5h). Raster plots demonstrated the decrease in burst number and spikes per bursts in the ASD co-cultures (Fig. 5j–l). These experiments suggest that the addition of healthy human astrocytes into mouse primary neuronal cultures (containing some mouse astrocytes) do not further support for network activity. However, we observe that the addition of ASD astrocytes influences functional properties of neurons including mean firing rate, synchronization, and spike numbers.

To determine whether ASD astrocytes alter structural plasticity, we quantified spine density in neurons co-cultured with CTRL or ASD astrocytes or no human astrocytes (None) (Fig. 5m). The presence of CTRL astrocytes did not significantly affect the number of spines on neurons when compared to None. However, the presence of ASD astrocytes decreased spine density on neurons when compared to CTRL and None groups (Fig. 5o).

These experiments complemented the behavioral and plasticity phenotypes of chimeric mice in supporting the conclusion that ASD astrocytes induce specific cognitive and behavioral deficits by influencing the structural and functional properties of neurons.

## Modulation of Ca^2+^ responses in ASD astrocytes

Astrocytes respond to neuronal activity via elevated cytosolic Ca^2+^ concentrations [69, 70]. This triggers the intracellular signaling pathways that modulate neuronal connectivity. Thus, exaggerated Ca^2+^ responses in ASD astrocytes could alter neuronal network activity and behavior.

**Fig. 6:**
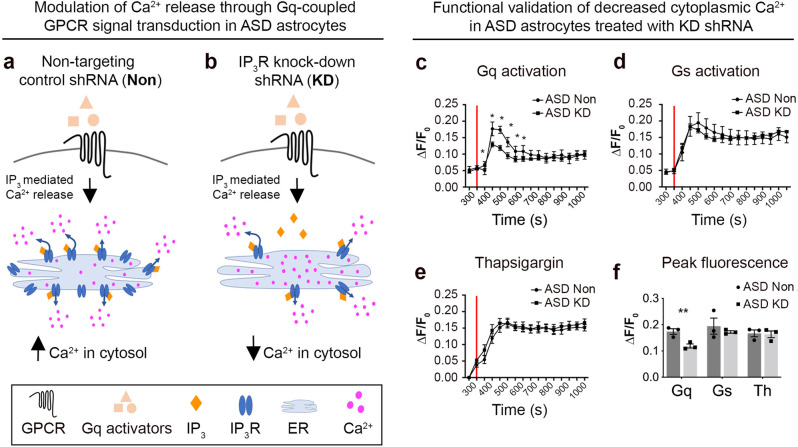
Modulation of Ca^2+^ signaling in ASD astrocytes. **a**, **b** ASD astrocytes were transduced with an shRNA lentivirus to downregulate IP_3_Rs (KD). As a control, ASD astrocytes were infected with a non-targeting shRNA lentivirus (Non). **c**–**f** A high throughput Ca^2+^ mobilization assay was optimized (see “Methods”) for validation of Ca^2+^ modulation upon knockdown of IP_3_Rs. **c** ASD KD astrocytes responded to the application of a cocktail of Gq activators (red line, 50 μM DHPG, 50 μM norepinephrine, 50 μM ATP, and 50 nM Endothelin 1) with diminished Ca^2+^ mobilization compared to ASD Non astrocytes. **d** ASD KD astrocytes did not display reduced Ca^2+^ mobilization in response to activation of Gs GPCR signal transduction (12.5 nM forskolin), which does not rely on IP_3_R mediated Ca^2+^ release from the ER, indicating the specificity of the KD system. **e** No differences were detected in the total Ca^2+^ concentration stored in the ER between ASD KD or Non astrocytes. Application of thapsigargin (2 μM, red line) depleted the ER Ca^2+^ and inhibited reuptake by Sarco/ER Ca^2+^-ATPase (SERCA) Ca^2+^ pumps. **f** ASD KD astrocytes showed lower Ca^2+^ responses to Gq activation compared ASD astrocytes, which indicates that IP_3_R reduction in ASD astrocytes modulated evoked increases in cytosolic Ca^2+^ from internal stores without changing its total concentration in the ER (Gq activation vs. Thapsigargin).

To reduce evoked Ca^2+^ increases, inositol 1,4,5-trisphosphate receptors (IP_3_R) type 1 and 2 were targeted using shRNA based knockdown strategy (Fig. 6 and see Supplementary Fig. 10 for knockdown validation as well as Methods and Key Resources table for further details). IP_3_Rs are ligand gated calcium channels found on the surface of the endoplasmic reticulum (ER) and upon ligand binding, IP_3_Rs release Ca^2+^ that is stored in high concentrations within the ER. Canonical activation occurs in response to Gq linked G protein coupled receptor (GPCR) signal transduction [71]. Gq activators bind to a GPCR leading to IP_3_ accumulation that targets IP_3_Rs on the ER. Thus, reducing IP_3_R levels reduces Ca^2+^ release from the ER [72] (schematic in Fig. 6a, b). We exploited the known role of IP_3_R-mediated Ca^2+^ release to commonly lower evoked elevations in cytosolic Ca^2+^ in ASD astrocytes. ASD astrocytes were infected with a shRNA lentivirus that targeted IP_3_Rs (KD). As a negative control, ASD or CTRL astrocytes were infected with a non-targeting shRNA lentivirus (Non).

To validate the reduction of Ca^2+^ mobilization in ASD KD astrocytes, a high throughput fluorescent screening assay in a 384-well format was optimized. This enabled application of stimulators under the same conditions with no time lag between wells. To activate G_q_, a cocktail of 50 μM DHPG, 50 μM norepinephrine, 50 μM ATP, and 50 nM Endothelin 1 was applied. Knockdown of IP_3_R in ASD astrocytes functionally reduced cytosolic Ca^2+^ upon activation of Gq signal transduction compared to ASD astrocytes treated with the non-targeting shRNA lentivirus (Fig. 6c). To activate G_s_, Forskolin (12.5 nM) and thapsigargin (2 μM) were delivered. Notably, activation of Gs linked GPCR signal transduction remained intact between ASD KD astrocytes and ASD Non astrocytes, indicating the specificity of the experimental system (Fig. 6d). Lastly, IP_3_R shRNA lentivirus did not affect the total Ca^2+^ concentration stored in the ER (Fig. 6e) as thapsigargin depleted ER Ca^2+^ stores and inhibited reuptake by SERCA Ca^2+^ pumps. Quantifications showed that ASD KD astrocytes had lower Ca^2+^ responses (shown with Gq activation) while same Ca^2+^ concentrations (thapsigargin application) compared to ASD Non astrocytes (Fig. 6f).

## ASD astrocytes with modulated Ca^2+^ signaling do not induce impaired network activity and memory

**Fig. 7:**
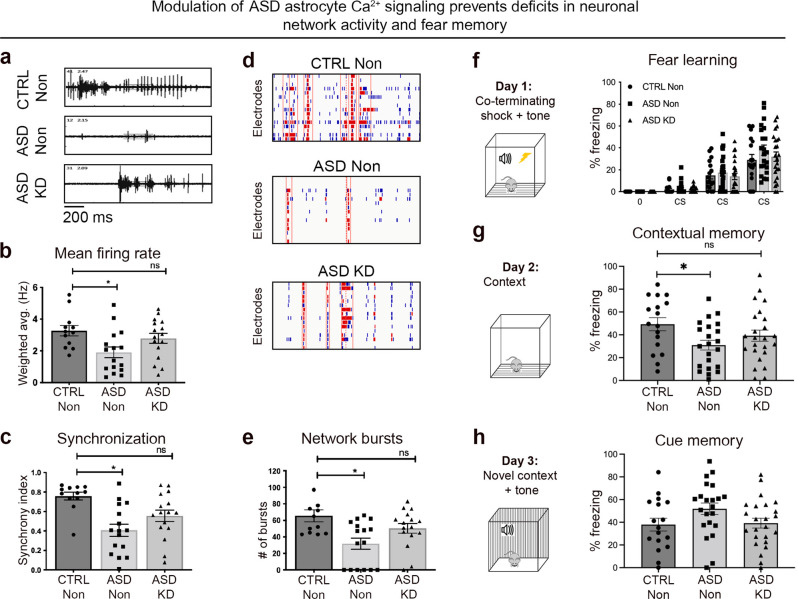
ASD astrocytes with modulated Ca^2+^ signaling do not induce impaired network activity and memory. **a**–**e** Human astrocytes were infected with either the non-targeting shRNA lentivirus (Non) or the IP_3_Rs shRNA lentivirus (KD) and co-cultured with primary hippocampal neurons. **a** Decrease in spiking pattern in untreated ASD Non co-cultures was corrected upon IP_3_Rs KD in ASD astrocytes. **b** As in Fig. 5, ASD co-cultures displayed decreased mean network firing rate when compared to CTRL co-cultures. Modulation of ASD astrocyte Ca^2+^ mobilization conferred protection against deficits in mean firing rate (see also Supplementary Video 3). **c** Similarly, ASD Non co-cultures displayed lower synchronicity that was corrected in ASD KD co-cultures. **d** Downregulation of IP_3_R rescued the phenotypes in network burst number (red hash marks surrounded by red boxes) and spikes per bursts (red hash marks) in the ASD astrocytes. **e** Unlike untreated ASD astrocytes, ASD KD astrocytes displayed similar number of network bursts when compared to CTRL astrocytes. These findings suggest that fine-tuning ASD astrocyte Ca^2+^ levels protects against deficits induced by untreated ASD astrocytes. **f**–**h** CTRL Non astrocytes, ASD Non astrocytes, and ASD KD astrocytes were transplanted into the brains of neonatal *Rag2*^*KO*^ mice. **f** Similar to the previous experiment, there was no significant difference in the rate of acquisition learning between CTRL and ASD mice. **g** As in Fig. 4, ASD Non chimeric mice showed reduced freezing behavior when exposed to the fear context relative to CTRL Non chimeric mice. However, this difference was eliminated between ASD KD and CTRL Non chimeric mice, indicating that amelioration of cytosolic Ca^2+^ in ASD astrocytes prevents fear memory deficits. **h** No significant differences were detected in freezing behavior between CTRL, ASD, and ASD KD mice during cue presentation in a novel context. Together, these results indicate that exaggerated Ca^2+^ release from internal stores in ASD astrocytes is responsible for neuronal network activity and memory deficits caused by these cells. MEA: CTRL Non co-cultures *n* = 12 wells, 3 distinct lines; ASD Non *n* = 10–16 wells, 4 distinct lines; ASD KD *n* = 15–16 wells, 4 distinct lines. Fear testing: CTRL Non *n* = 17 male and female mice, transplanted with 2 distinct lines, ASD Non *n* = 22 male mice, transplanted with 4 distinct lines, ASD KD *n* = 24 male mice, transplanted with 4 distinct lines. CTRL Non: Control human astrocytes infected with non-targeting shRNA lentivirus. ASD Non: ASD astrocytes infected with non-targeting shRNA lentivirus. ASD KD: ASD astrocytes infected with IP_3_R shRNA lentivirus.

Next, we tested whether attenuation of Ca^2+^ mobilization prevented dysfunction induced by ASD astrocytes. WT hippocampal neurons were co-cultured with Non or KD shRNA infected ASD astrocytes. Similar to the experiments in Fig. 5, ASD Non astrocytes decreased mean firing rate, synchronization, and total number of network bursts in WT hippocampal neurons when compared to CTRL Non astrocytes (Fig. 7b–e). Compared to CTRL Non astrocyte co-cultures, mean firing rate, synchronization, and total number of network bursts were not significantly different in ASD KD astrocyte co-cultures suggesting that attenuation of cytosolic Ca^2+^ levels has a protective effect (Supplementary Video 3). Representative raw traces of spiking pattern (Fig. 7a) and raster plots of network bursts (Fig. 7d) visually illustrated that reduced cytosolic Ca^2+^ levels in ASD KD astrocytes had a protective effect on spike and synchronized bursting activity in primary neurons relative to ASD Non astrocytes with high cytosolic Ca^2+^ levels.

To determine if ASD KD astrocytes induced fear memory deficits in chimeric mice similar to ASD astrocyte chimeric mice, ASD astrocytes infected with IP_3_R KD shRNA or Non shRNA lentivirus were transplanted into neonatal *Rag2*^*KO*^ mice. Hippocampal learning and memory were compared in three groups of mice: (1) CTRL Non astrocyte chimeric mice, (2) ASD Non astrocyte chimeric mice, and (3) ASD KD astrocyte chimeric mice. Similar to the finding in Fig. 4, all three groups acquired the association between the audible tone and the mild electrical foot shock at the same rate (Fig. 7f). Consistent with the previous result, ASD Non astrocyte chimeric mice displayed deficits in contextual fear memory relative to CTRL Non astrocyte chimeric mice (Fig. 7g). The attenuated fear memory phenotype was not present in ASD KD chimeric mice as these mice froze at a similar rate compared to CTRL Non chimeric mice during memory assessment on 2nd day of the test (Fig. 7g).

Together, these results suggest that altered Ca^2+^ signaling in ASD astrocytes contribute to impaired network activity and memory in ASD chimeric mice.

## Discussion

Our study identifies aberrant Ca^2+^ signaling in ASD astrocytes as a mechanism that contributes to specific behavioral and neuronal deficits. The results of this study provide insight into how astrocyte dysfunction might contribute to behavioral phenotypes of ASD and advances our understanding of disease pathogenesis.

Data in this study demonstrate that ASD astrocytes induce repetitive behavior as well as deficits in memory and synaptic plasticity, which reflects changes in neuronal network dynamics. To determine disease-specific properties inherent to ASD astrocytes, we isolated astrocytes from iPSC-derived organoids and expanded them in astrocyte selection media (Fig. 1). When these ASD astrocytes were transplanted into postnatal brains, ASD chimeric mice exhibited perseverative digging behavior (Fig. 4g, h) and attenuated fear memory (Fig. 4a–d). There is a male bias in ASD prevalence [57]. However, the sex split analyses showed that the behavioral deficits caused by ASD astrocytes were not dependent on the sex of chimeric mice (Supplementary Fig. 7). In an independent experiment, where neural progenitor cells (NPCs) from other ASD iPSC lines were transplanted into neonatal brains, a defective memory phenotype was recapitulated (Supplementary Figs. 11–13). The vast majority of CTRL and ASD NPCs terminally differentiated into astrocytes in the adult chimeric brains (Supplementary Fig. 12). Together, this suggests that ASD astrocytes causally induce behavioral deficits in chimeric mice.

We also found that ASD astrocyte chimeric mice manifest altered LTP relative to CTRL chimeric mice (Fig. 4e, f). Astrocytes may deeply impact neuronal function through the release of gliotransmitters. In fact, a prior study demonstrated that astrocytic release of ATP suppresses synaptic transmission and regulates the dynamic range for LTP generation [73]. Likewise, astrocytes have been implicated in the formation of new memories through modulating LTP [21], and experimental manipulations of LTP can induce behavioral impairments associated with ASD [74, 75]. LTP is composed of two different phases [61]: E-LTP and L-LTP. Intriguingly, transplantation of ASD astrocytes specifically disrupted E-LTP. Determining the molecular and cellular mechanism by which astrocytes impact distinct phases of LTP requires further investigation. Nevertheless, our results indicate that ASD astrocyte transplantation negatively impacts LTP and ultimately behavior.

For chimera studies, use of immunocompromised mice as graft hosts is necessary to avoid rejection [39]. Specifically, T-cell and B-cell development is arrested in *Rag2*^KO^ mice. Most studies assessing B-cell number and function did not detect any abnormalities in ASD cases [76]. A study found higher number of T-lymphocytes as well as microscopic blebs associated with astrocytes in the perivascular space in postmortem ASD brains when compared to a control group [77]. However, whether this dysregulation is primary or secondary to the disease is not clear. While immune system integrity could be a contributing factor to ASD pathogenesis [78], it is unlikely the sole factor responsible for all ASD behavioral phenotypes. Our data provide evidence that ASD astrocytes induce behavioral and synaptic plasticity deficits in chimeric mice, suggesting a causal contribution of astrocytes to the disease independent of compensatory responses that could be present in the ASD brain.

It has been posited that the inability of the ASD brain to properly synchronize neuronal activity contributes to behavioral impairments [79, 80]. Here, we show that ASD astrocytes cause reduced spine density and network activity in hippocampal neurons in vitro. Neuronal connectivity and spine densities are intimately dependent on astrocyte function [14, 81]. Further, aberrant spine densities were detected in ASD postmortem brains [82–84]. Spine density most likely operates in an optimal range to subserve proper cognitive function; thus too many or too few spines is deleterious [85]. Accompanying the reduced spine phenotype (Fig. 5m–o), we also found reduced network and synchronous activity in neurons co-cultured with ASD astrocytes compared to those co-cultured with CTRL astrocytes (Fig. 5a–l). These experiments support the conclusion that ASD astrocytes induce specific cognitive and behavioral deficits by influencing the structural and functional properties of neurons.

Results here identify exaggerated evoked Ca^2+^ signaling as an inherent mechanism that contributes to ASD astrocyte-mediated phenotypes. Proteins that serve in Ca^2+^ signaling are differentially regulated in ASD astrocytes when compared to CTRL astrocytes (Fig. 1b–e). Two-photon Ca^2+^ imaging both in vitro (Fig. 1f–l) and in transplanted cells in vivo (Fig. 3) confirmed aberrant Ca^2+^ mobilization within several distinct ASD iPSC-derived astrocytes compared to CTRL astrocytes. In vivo Ca^2+^ imaging of transplanted human astrocytes in awake behaving mice showed that ASD astrocytes exhibited significantly heightened responses to environmental stimuli. Recent sequencing studies suggest distinct mutations cluster on neural communication and plasticity networks [86–88]. Consistent with these findings, it was possible to correct deficits in multiple ASD mouse models by targeting common signaling hubs critical for plasticity [74, 89]. Because each ASD line may exhibit varied mechanisms for increased cytosolic Ca^2+^, lowering IP_3_R levels would result in a common reduction in Ca^2+^ release from the ER across all ASD-derived astrocytes. When evoked Ca^2+^ responses were attenuated by downregulation of IP_3_Rs in ASD astrocytes prior to transplantation, the fear memory phenotype was partially rescued in ASD astrocyte chimeric mice (Fig. 7f–h). We also show that attenuation of Ca^2+^ mobilization in ASD astrocytes in co-cultures prevented all neuronal network activity phenotypes previously observed in ASD astrocytes and hippocampal neuron co-cultures (Fig. 7a–e and see also Supplementary Video 3). Full genetic knockout of IP_3_Rs eliminates nearly all somatic Ca^2+^ transients, while having no effect on neuronal excitability, synaptic currents, or synaptic plasticity [90]. Thus, modulation of Ca^2+^ signaling with this system reduces non-specific adverse effects to general electrophysiology properties. While IP_3_ receptors 1 and 2 are well-known to regulate cytoplasmic Ca^2+^ levels, we cannot discard the possibility that their downregulation could cause phenotypes beyond Ca^2+^ signaling.

## Future directions

Astrocytes represent a population of complex and functionally diverse cells [91]. We observed that the engrafted astrocytes exhibited morphologies that resemble protoplasmic astrocytes. However, the heterogeneity of astrocytes isolated from organoids and the role of distinct astrocyte subpopulations in ASD remains unclear. An interesting future extension of this work is the application of single-cell RNA sequencing (scRNA-seq) to engrafted mature CTRL and ASD astrocytes. scRNA-seq enables comparison of transcriptomes of individual cells. Thus, in addition to assessing transcriptional similarities and differences within cell populations, scRNA-seq also reveals cell heterogeneity.

Understanding astrocyte heterogeneity is particularly important because specific populations may influence distinct aspects of neuronal function. In this regard, we observed that ASD astrocytes negatively influence E-LTP and spine density. In the initial phase of LTP, the increase in both number and single-channel conductance of postsynaptic AMPAR leads to an enhancement of synaptic transmission. Elevated astrocytic Ca^2+^ was reported to influence the release of gliotransmitters such as glutamate, ATP, D-serine, and GABA [69, 70, 92]. Alterations in gliotransmitter release may negatively influence AMPAR insertion into the membrane and thereby lead to reductions in E-LTP. We found no difference in glutamate levels between ASD astrocytes and CTRL astrocytes (Supplementary Fig. 14). However, the gliotransmitters released by ASD astrocytes and their impact on synaptic and structural plasticity requires further investigation. Of note, it was reported that astrocytic ATP release, which is regulated by Ca^2+^ activity in astrocytes, is required for astrocyte-mediated synapse elimination [93]. Thus, dysregulated ATP release in ASD astrocytes could account for the spine density phenotype caused by these cells. Astrocytes are known to mediate synapse elimination through phagocytosis [94]. Intriguingly, the IPA of CTRL and ASD astrocyte proteome indicated alterations in actin filament binding, structural constituents of the cytoskeleton, and myosin phosphatase activity in ASD astrocytes (Fig. 1b). Assessing motility as well as phagocytic activity of ASD astrocytes could reveal an underappreciated role of astroglia in neurodevelopmental disorders.

Here, we prioritized behavioral tests pertinent to sociability, perseveration and memory. In future studies, this initial characterization could be elaborated to assess more complex types of cognitive function, such as reversal learning in ASD astrocyte chimeric mice.

An important limitation in our study is controlling the precise ratio of engrafted human astrocytes to native mouse astrocytes in chimeric mice and co-culture assays. Determining the minimum number of ASD astrocytes that is sufficient to cause behavioral or neuronal phenotypes may be useful for “fine-tuning” these model systems. Finally, in our co-culture assays we aimed to mimic the in vivo environment by culturing ASD astrocytes with mouse hippocampal neurons rather than human iPSC-derived neurons. Our co-culture experimental design complemented in vivo experiments in supporting the conclusion that ASD astrocytes elicit synaptic plasticity and behavioral deficits by influencing structural and functional properties of neurons. How iPSC-derived human astrocytes influence human neuronal maturation and functional properties in culture is controversial [95, 96] and is yet to be established for neuronal network activity. However, assessing how human ASD astrocytes influence control iPSC- or ASD iPSC-derived neurons might be valuable to provide further insight into pathophysiological mechanisms of ASD.

## Methods

### Induced pluripotent stem cells (iPSCs)

iPSC lines were purchased from NIH and CIRM repositories (Supplementary Table 1, see also Key Resources table in Supplementary Methods). Please see Supplementary Tables 1–3 for clinical as well as genetic information. Key resources table includes information for purchasing. Supplementary Table 4 provides a list of experiments and specific lines used for each experiment. See Supplementary Methods for further details for iPSCs.

Methodological details of all experiments as well as detailed descriptions of specific experiments can be found in Supplementary Information document. Please see Supplementary Information for acknowledgements and author contributions.

## Supplementary information

Supplemental
