# Supplementary material for: Astrocytes derived from ASD individuals alter behavior and destabilize neuronal activity through aberrant Ca2+ signaling
Source: Mol Psychiatry. 2022 Apr 1;27(5):2470–84. doi: 10.1038/s41380-022-01486-x (PMC9135629; doi:10.1038/s41380-022-01486-x)
Supplement: Supplementary file 1 — Supplemental [file 41380_2022_1486_MOESM1_ESM.pdf]

Supplementary Information

Astrocytes derived from ASD individuals alter behavior and destabilize neuronal activity through aberrant Ca^{2+} signaling

Megan Allen[1,10], Ben S. Huang[1,8,10], Michael J. Notaras[1], Aiman Lodhi[1], Estibaliz Barrio Alonso[1], Pablo J. Lituma[1], Paul Wolujewicz[1], Jonathan Witztum[1], Francesco Longo[2], Maoshan Chen[3-6], David W. Greening[3-6], Eric Klann[2], M. Elizabeth Ross[1], Conor Liston[1,8], Dilek Colak[1,7,9]

[1] Center for Neurogenetics, Feil Family Brain and Mind Research Institute, Weill Cornell Medicine, Cornell University, New York, USA.
[2] Center for Neural Science, New York University, New York, USA.
[3] Molecular Proteomics, Baker Heart & Diabetes Institute, Melbourne, Australia.
[4] Baker Department of Cardiometabolic Health, The University of Melbourne, Australia
[5] Baker Department of Cardiovascular Research, Translation and Implementation, La Trobe University, Australia
[6] Central Clinical School, Monash University, Australia
[7] Gale & Ira Drukier Institute for Children's Health, Weill Cornell Medicine, Cornell University, New York, USA.
[8] Department of Psychiatry, Weill Cornell Medicine, Cornell University, New York, USA.

[9]Correspondence: dic2009@med.cornell.edu
[10]These authors contributed equally to this work

Supplementary Information

Supplementary information includes 4 tables, 14 figures, 3 videos, methods, acknowledgements, and author contributions.

Supplementary Tables

Supplementary Table 1: Information about CTRL and ASD lines

Supplementary Table 2: Counts of hits for LGD SNVs and LGD CNVs in CTRL and ASD individuals

Supplementary Table 3: Variants in CTRL and ASD individuals and which individual carries them

Supplementary Table 4: CTRL and ASD lines used in each experiment

Supplementary Figures

Supplementary Fig. 1, Related to Fig. 1: Organoid-derived astrocytes express astrocyte markers.

Supplementary Fig. 2, Related to Fig. 1: Astrocytes dissociated from organoids and maintained in astrocyte selection media are not overtly reactive.

Supplementary Fig. 3, Related to Fig. 1: Detection of astrocyte specific proteins through proteomic analysis**.**

Supplementary Fig. 4, Related to Fig. 2: No significant difference in human astrocyte numbers between CTRL and ASD astrocyte chimeric brains.

Supplementary Fig. 5, Related to Fig. 2: Transplanted human cells do not terminally differentiate to neurons in chimeric brains.

Supplementary Fig. 6, Related to Fig. 4: General motor activity is similar between ASD chimeric and CTRL chimeric mice.

Supplementary Fig. 7, Related to Fig. 4: Sex split analyses of fear memory and marble burying performance in chimeric mice.

Supplementary Fig. 8, Related to Fig. 4: Spatial learning, spatial memory and sociability are not impaired in ASD astrocyte chimeric mice.

Supplementary Fig. 9, Related to Fig. 5: Characterization of mouse hippocampal neuronal cultures.

Supplementary Fig. 10, Related to Fig.6: Validation of IP_3R downregulation.

Supplementary Fig. 11, Related to Discussions: iPSCs derived from ASD or CTRL individuals uniformly convert to NPCs.

Supplementary Fig. 12, Related to Discussions: Neural progenitors derived CTRL and ASD iPSCs terminally differentiate to astrocytes upon transplantation into the mouse brain.

Supplementary Fig. 13, Related to Discussions: ASD NPC chimeric mice display impaired associative memory but not learning deficits.

Supplementary Fig. 14, Related to Discussions: Glutamate levels are not significantly different between CTRL and ASD astrocytes.

Supplementary Videos (Provided as separate files)

Supplementary Video 1, Related to Fig. 1: ASD astrocytes respond to stimulation with more intense Ca^{2+} transients compared to CTRL astrocytes.

Supplementary Video 2, Related to Fig. 5: ASD astrocyte co-cultures display decreased mean network firing rate when compared to CTRL co-cultures and a control group that contains only dissociated mouse neurons.

Supplementary Video 3, Related to Fig. 7: Modulation of ASD astrocyte Ca^{2+} mobilization confers protection against deficits in network firing recorded with MEA.

Supplementary Table 1: Information about CTRL and ASD iPSC lines

ID	Information
CW20008	ASD; male, Asian, sample collected at age 5, low IQ (68), communication and social deficits (ADOS score 16) as well as stereotyped behaviors and restricted interests (ADOS score 5)
CW20142	ASD; male, Asian, sample collected at age 9, restricted and repetitive behavior (ADOS score 2), communication and social deficits (ADOS score 9)
CW20081	ASD; male, White, sample collected at age 15, low IQ (40), poor eye contact, communication and social deficits (ADOS score 18) as well as stereotyped behaviors and restricted interests (ADOS score 5)
CW20083	ASD; male, White, sample collected at age 22, poor eye contact, severe communication and social deficits (ADOS score 18) repetitive behavior (ADOS score 8)
CW60115	ASD; male, White, sample collected at age 4, poor eye contact, social deficits, stereotyped behaviors and restricted interests (ADOS score 3)
MH0148698	ASD; male, White, low IQ, severely affected (Mariani *et al*., 2015)
MH0148713	ASD; male, White, low IQ, severely affected (Mariani *et al*., 2015)
CW20026	ASD; male, White, sample collected at age 10, low IQ (52), communication and social deficits (ADOS score 19) as well as stereotyped behaviors and restricted interests (ADOS score 3)
CW20044	ASD; male, White, sample collected at age 10, low IQ (40), spinning, severe communication deficits (ADOS score 16) as well as stereotyped behaviors and restricted interests (ADOS score 7)
MH0159019	CTRL, female, Hispanic, sample collected at age 29
MH0159020	CTRL, male, White, sample collected at age 58
MH0159021	CTRL, male, Hispanic, sample collected at age 32
MH0174677	CTRL, male, White, male, white, sample collected at age 9
MH0174679	CTRL, male, White, male, white, sample collected at age 17
MH0174681	CTRL, male, White, male, white, sample collected at age 8
MH0174686	CTRL, male, White, sample collected at age 17
GM23279	CTRL, female, White, sample collected at age 36
GM25256	CTRL, male, Asian, sample collected at age 30

Supplementary Table 2: Summary stats for LGD SNVs and LGD CNVs in CTRL and ASD individuals

	CTRL (9 lines)	ASD (9 lines)
Rare (< 0.01 MAF), Likely Gene-Disrupting (LGD) SNVs/Indels (322 ASD gene panel)	37	33
Rare (< 0.01 MAF), Likely Gene-Disrupting (LGD) CNVs Overlapping ClinVar P/LP	31	25

Supplementary Table 3: Variants in CTRL (37) and ASD (33) lines and which individual carries them

Chromosome and gene	Start	End	Alternate Allele	Sample
chr1; RERE	8656247	8656247	CGGTCC	ASD; CW20026_S55
chr1: TEKT2	36088007	36088007	T	ASD; CW20083_S56
chr10; ANK3	60042719	60042719	T	ASD; CW20026_S55
chr11; SRPRA	126265815	126265815	T	ASD; CW60115_S58
chr15; MAP1A	43525917	43525917	A	ASD; CW20044_S54
chr15; MAP1A	43527664	43527664	T	ASD; CW20044_S54
chr16; TSC2	2053391	2053391	T	ASD; CW20081_S59
chr16; TEKT5	10635894	10635894	G	ASD; CW60115_S58
chr16; SETD1A	30964952	30964952	T	ASD; CW20044_S54
chr17; TEKT1	6819266	6819266	C	ASD; CW20026_S55
chr19; ZNF180	44476523	44476523	A	ASD; MH0148713_S63
chr2; MYT1L	1943258	1943258	T	ASD; CW20142_S61
chr2; RNF181	85595845	85595845	T	ASD; CW20142_S61
chr2; MBD5	148489641	148489641	G	ASD; CW20008_S57
chr3; SETD5	9464601	9464601	C	ASD; CW20008_S57
chr3; NBEAL2	47004352	47004352	T	ASD; CW20083_S56
chr3; SETD2	47121443	47121443	A	ASD; CW20142_S61
chr3; CACNA2D3	54891399	54891399	T	ASD; MH0148713_S63
chr4; ANK2	113354794	113354794	T	ASD; MH0148713_S63
chr4; NR3C2	148154604	148154604	T	ASD; CW20081_S59
chr5; ACTBL2	57481701	57481701	T	ASD; CW20142_S61
chr7; RELN	103539111	103539111	T	ASD; CW20083_S56
chr7; RNF133	122698017	122698017	G	ASD; CW20026_S55
chr7; KMT2C	152205112	152205112	G	ASD; CW60115_S58
chr8; CHD7	60865328	60865328	T	ASD; CW20026_S55
chr8; ZNF16	144931535	144931535	C	ASD; CW20142_S61
chr1; B4GALT3	161173722	161173722	A	ASD; MH0148713_S63
chr16; CDH15	89192318	89192318	C	ASD; CW20008_S57
chr17; PIGL	16317813	16317813	T	ASD; CW20083_S56
chr18; CNDP2	74519021	74519021	T	ASD; CW20142_S61
chr19;COLGALT1	17577383	17577383	A	ASD; CW20008_S57
chr22; A4GALT	42693060	42693060	A	ASD; CW20142_S61
chr5; NSUN2	6600170	6600170	T	ASD; CW20026_S55
chr20; ARFGEF2	49017279	49017279	C	CTRL; MH0159020_S9
chr1; A3GALT2	33307191	33307191	T	CTRL; MH0174677_S7
chr1; A3GALT2	33307415	33307415	A	CTRL; MH0159019_S5
chr1; DPYD	97573870	97573870	T	CTRL; MH0174681_S3
chr1; GJA8	147908613	147908613	G	CTRL; MH0174681_S3
chr1; PDE4DIP	149021033	149021033	A	CTRL; GM23279_S2
chr1; ASH1L	155478027	155478027	C	CTRL; MH0159019_S5
chr1; CACNA1E	181776219	181776219	A	CTRL; MH0159019_S5
chr1; CACNA1E	181783712	181783712	A	CTRL; MH0174677_S7
chr10; PCDH15	54213995	54213995	A	CTRL; MH0159020_S9

chr10; ANK3	60042719	60042719	T	CTRL; GM23279_S2
chr11; CLP1	57659760	57659760	G	CTRL; MH0159019_S5
chr11; SHANK2	70490374	70490374	T	CTRL; MH0159019_S5
chr13; NBEA	35665135	35665135	T	CTRL; MH0174679_S2
chr16; CASKIN1	2179616	2179616	A	CTRL; MH0174677_S7
chr16; CASKIN1	2181024	2181024	C	CTRL; MH0174677_S7
chr16; TEKT5	10694767	10694767	C	CTRL; MH0174681_S3
chr17; RNF135	30999137	30999137	T	CTRL; MH0174677_S7
chr18; ASXL3	33739587	33739587	A	CTRL; MH0159020_S9
chr19; PRR12	49597037	49597037	G	CTRL; MH0159021_S6
chr19; ZNF175	51587688	51587688	A	CTRL; GM23256_S8
chr2; BAZ2B	159412481	159412481	T	CTRL; MH0174679_S2
chr2; SCN2A	165367225	165367225	T	CTRL; MH0159019_S5
chr2; SCN1A	166073486	166073486	T	CTRL; MH0174681_S3
chr2; ERFE	238165677	238165677	G	CTRL; MH0159021_S6
chr3; SETD5	9464601	9464601	C	CTRL; GM23256_S8
chr3; SETD2	47121346	47121346	C	CTRL; MH0174686_S1
chr3; RNF123	49699104	49699104	T	CTRL; GM23256_S8
chr3; PCCB	136301086	136301086	G	CTRL; MH0159020_S9
chr5; SLC6A18	1244318	1244318	T	CTRL; MH0159019_S5
chr5; GABRB2	161330900	161330900	A	CTRL; GM23256_S8
chr6; HIVEP2	142774335	142774335	C	CTRL; GM23279_S2
chr6; ARID1B	156778665	156778665	A	CTRL; MH0159019_S5
chrX; NEXMIF	74740232	74740232	A	CTRL; MH0174686_S1
chrX; PCDH19	100296404	100296404	T	CTRL; GM23256_S8
chrX; FLNA	154353346	154353346	A	CTRL; MH0174686_S1
chr1; RERE	8361046	8361046	GGGATGCGGCGG	CTRL; MH0159021_S6

Supplementary Table 4: CTRL and ASD iPSC lines used in each experiment

Experiment	Lines
Whole Exome Sequencing (Supp Table 2 and 3)	All lines in Supp table 1 (9 ASD, 9 CTRL)
Proteomics (Fig.1 and Supp Fig. 3)	All lines in Supp table 1 except a CTRL line MH0159019 (9 ASD, 8 CTRL)
Astrocyte reactivity (Supp Fig. 2)	All lines in Supp table 1 (9 ASD, 9 CTRL)
In vitro Ca^{2+} imaging (Fig. 7)	CTRL: MH0159020, MH0159021, MH0174677, MH0159019, GM25256; ASD: CW20008, CW20083, CW60115, CW20142, CW20081
MEA (Fig. 5)	CTRL: MH0159020, MH0159021, MH0159019 ASD: CW20008, CW20083, CW60115, CW20081
Spine quantifications (Fig. 5)	CTRL: MH0159020, MH0159021, MH0174677, MH0159019; ASD: CW20008, CW20083, CW60115, CW20142, CW20081
Stereological Quantifications (Supp Fig. 4)	CTRL: MH0159020, MH0159021, MH0174677 ASD: CW20008, CW20083, CW60115
NeuN+GFP+ co-localization in chimeric brains (Supp Fig. 5)	CTRL: MH0159020, MH0159021, MH0174677, MH0159019; ASD: CW20008, CW20083, CW60115, CW20142, CW20081
Live animal Ca^{2+} imaging (Fig. 3)	CTRL: GM23256, MH0159020, MH0159021, MH0174677; ASD: CW20008, CW20083, CW20142, CW20081
Behavior or LTP experiments in astrocyte chimeric mice (Fig. 4)	CTRL: MH0159019, MH0159020, MH0159021, MH0174677, GM25256; ASD: CW20008, CW20142, CW20081, CW20083, CW60115
MWM or Sociability (Supp Fig. 8)	CTRL: MH0159019, MH0159020, MH0159021, MH0174677; ASD: CW20008, CW20081, CW60115
Rescue- MEA (Fig. 7)	CTRL: MH0159020, MH0159021, MH0174677 ASD: CW20083, CW60115, CW20142, CW20081
Rescue- Fear Conditioning (Fig. 7)	CTRL: MH0159021, MH0159019 ASD: CW20083, CW60115, CW20142, CW20081
NPC experiments (Supp Fig. 11-13)	CTRL: MH0159019, MH0159021, MH0159021; ASD: CW20008, CW60115, CW20142, CW20083
Glutamate Assay (Supp Fig. 14)	All lines in Supp table 1 except female CTRL lines MH0159019 (9 ASD, 7 CTRL)

Supplementary Fig. 1

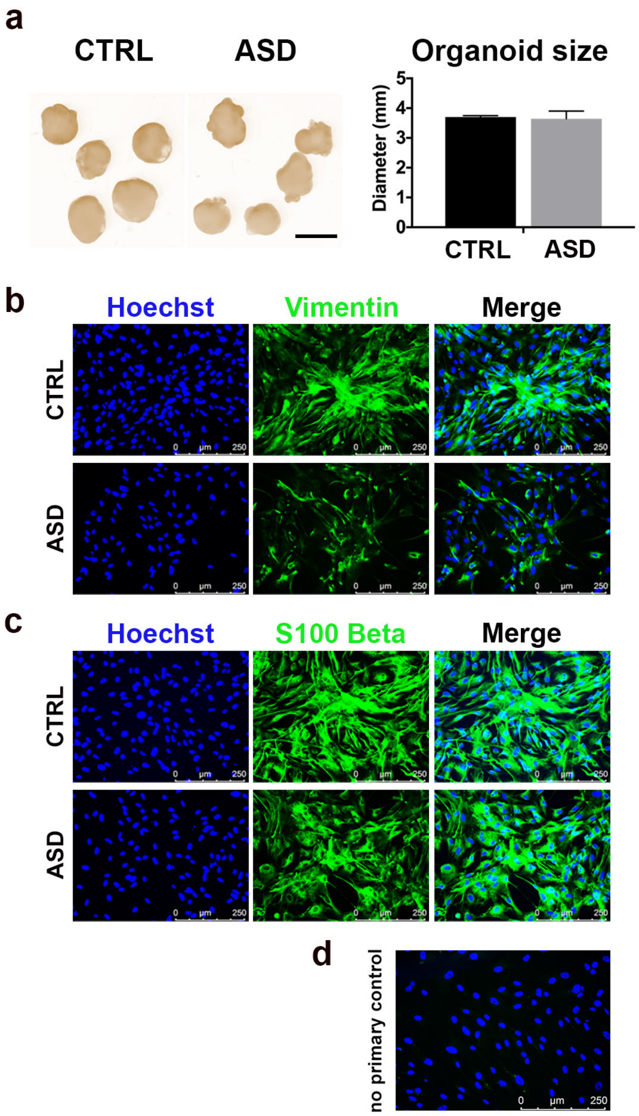

Supplementary Fig. 1: Organoid-derived astrocytes express astrocyte markers, related to Fig. 1.

a, Representative images of CTRL and ASD cerebral organoids at day 70.

b,c, Representative images from immunostainings showed that cells dissociated from organoids (at day 75) expressed Vimentin (**b**) and S100 Beta (**c**), two well-described astrocyte markers (see also **Fig. 1** and **Supp Fig. 3** for additional markers) **d,** Representative no-primary antibody control immunostained image demonstrated the specificity of positive signal in b and c. No-primary control was used to determine imaging exposure times. Scale bar = 250 μm.

Supplementary Fig. 2

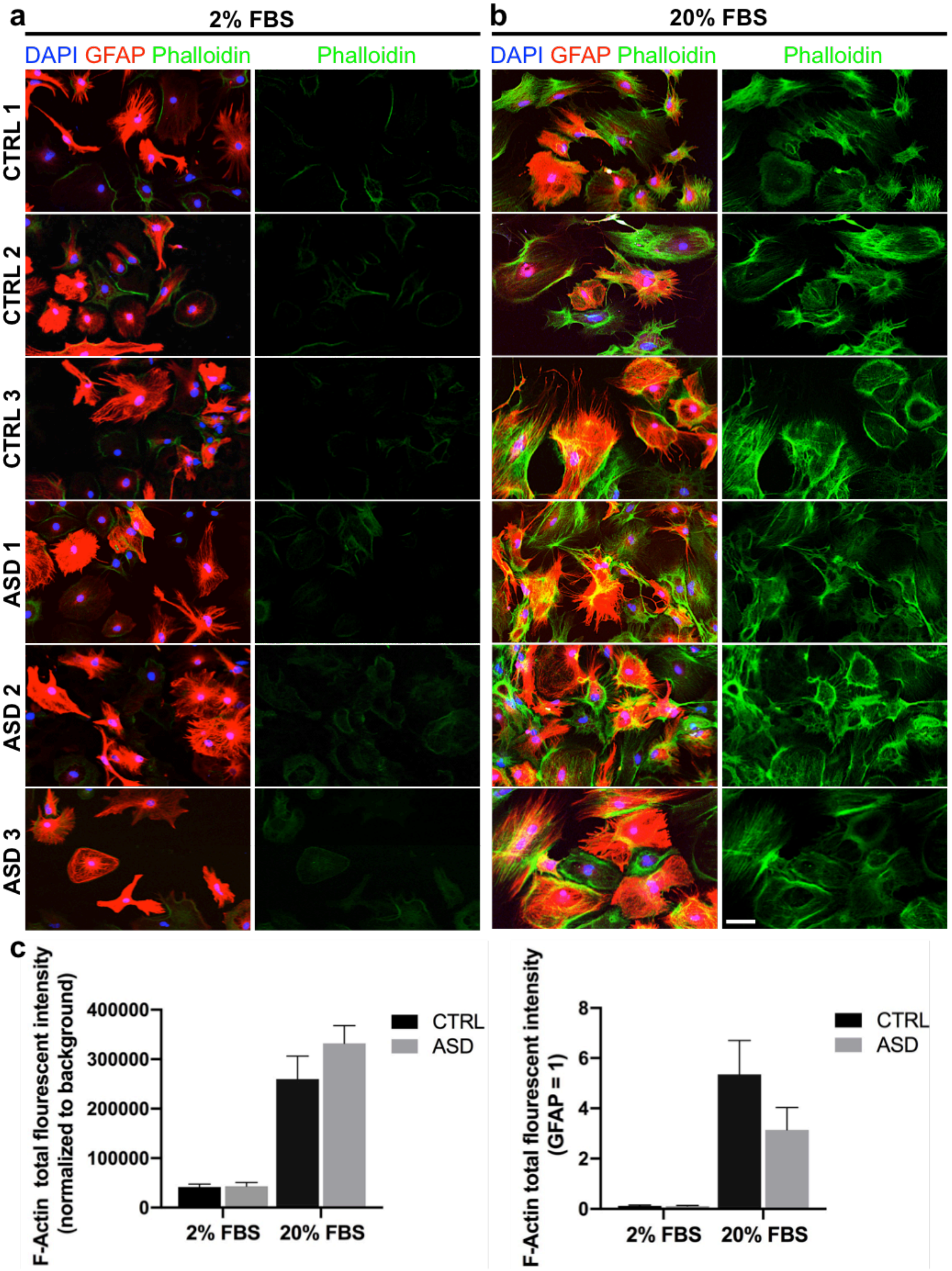

Supplementary Fig. 2: Astrocytes dissociated from organoids and maintained in astrocyte selection media are not overtly in reactive state, related to Fig. 1.
To assess whether organoid-derived CTRL or ASD astrocytes were reactive, we visualized F-Actin by Phalloidin labeling and quantified F-Actin fluorescent intensity. Our astrocyte selection media contains minimal FBS (2%) that is required for astrocyte proliferation and known to cause no reactivity in these cells. Because high levels of FBS is known to induce reactivity in astrocytes, we cultured both CTRL and ASD organoid derived astrocytes in astrocyte selection media with 20% FBS, as a positive control, only in this experiment. Quantifications in **c** were done in astrocytes generated from all CTRL (9 distinct iPSC lines) and ASD (9 distinct iPSC lines) lines used in this paper.

In addition, we also assessed the reactivity status of our astrocytes by screening for specific reactive astrocyte markers within our astrocyte proteomics datasets. For this, we consulted two papers[1,2] that together identified 12 markers whose levels were increased only when astrocytes are reactive. Of these, 9 reactive astrocyte markers *were not detected* via TMT-LC/MS proteomics in any of our CTRL or ASD samples (SERPINA3N, TNFRSF12A, S1PR3, CXCL1, CXCL2, CXCL10, LCN2, STEAP4, and OSMR). 3 reactive astrocyte markers (PTX3, TIMP1, and CD44) maintain other homeostatic cellular activities and were identified in our samples as expected. However, expression levels of these remaining factors were relatively low in abundance and without any significant p value or Log2FC value change.

a,b, Representative images of astrocytes (isolated from organoids derived from n = 3 distinct iPSCs per group) that were cultured in 2% (**a**) or 20% (**b**) FBS, and stained with GFAP and Phalloidin. While F-Actin signal can be seen at similar and relatively low levels in both CTRL and ASD astrocytes in our regular culture conditions (2% FBS), the F-Actin signal is highly increased in these cells when cultured in reactivity-inducing conditions, i.e., 20% FBS. **c,** We measured total F-Actin fluorescent in astrocytes generated from all CTRL and ASD iPSC lines used in the paper (n = 9 distinct ASD lines, n = 9 distinct CTRL lines). In both quantifications when either normalized to background (left) or, as an internal control, to GFAP (right), F-Actin levels in both group found to be significantly increased in reactivity-inducing conditions.

Together, these data suggest that CTRL and ASD organoid-derived astrocytes that are maintained in astrocyte selection media are not in a reactive state.
Scale bar = 50 μm Data are represented as mean ± SEM. n = 9 distinct ASD lines, n = 9 distinct CTRL lines.

Supplementary Fig. 3

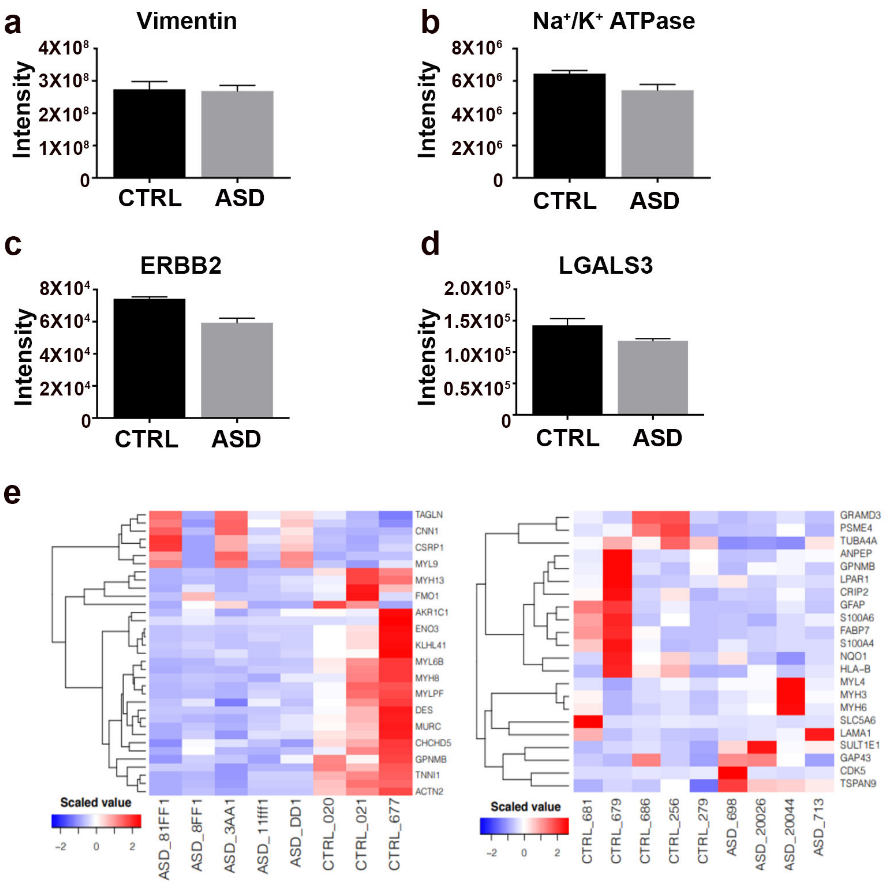

f

Protein IDs	Gene names	Protein names	log2 FC (ASD/Ctrl)	p-value	Regulation (ASD/Ctrl)
Q96A32	MYLPF	Myosin regulatory light chain 2, skeletal muscle isoform	-3.555	1.25E-07	down-regulated
P63316	TNNC1	Troponin C, slow skeletal and cardiac muscles	-2.953	7.67E-06	down-regulated
P35609	ACTN2	Alpha-actinin-2	-2.001	6.26E-03	down-regulated
P17302	GJA1	Gap junction alpha-1 protein	-1.918	8.81E-03	down-regulated
P19237	TNNI1	Troponin I, slow skeletal muscle	-1.893	1.29E-02	down-regulated
P21980	TGM2	Protein-glutamine gamma-glutamyltransferase 2	1.534	3.24E-02	up-regulated
Q01995	TAGLN	Transgelin	1.788	6.15E-03	up-regulated
P24844	MYL9	Myosin regulatory light polypeptide 9	1.965	1.60E-03	up-regulated

Protein IDs	Gene name	Protein names	log2 FC (ASD/Ctrl)	p-value	Regulation (ASD/Ctrl)
P06703	S100A6	Protein S100;Protein S100-A6	-2.407	3.15E-05	down-regulated
P26447	S100A4	Protein S100-A4	-1.965	9.00E-04	down-regulated
Q92633	LPAR1	Lysophosphatidic acid receptor 1	-1.561	2.22E-02	down-regulated
Q00535	CDK5	Cyclin-dependent-like kinase 5	2.005	1.24E-03	up-regulated

Supplementary Fig. 3: Detection of astrocyte specific proteins through proteomic analysis, related to Fig. 1.

To identify the inherent pathological properties within ASD astrocytes, we took an unbiased approach and compared the protein profiles of 9 ASD patient astrocytes to 8 healthy CTRL subject astrocytes with Tandem mass tag (TMT) liquid chromatography mass spectrometry.

a-d, Proteomic analysis detected several markers for astrocyte identity in our samples and the intensities are compared in the graphs.

e,f, Expression based heat maps from two independent experiments exhibited quantitative patterns across proteins and biological samples (CTRL n = 8 unique lines and ASD n = 9 unique lines). Each column represents astrocytes from a unique line, while relevant proteins are represented in each row. (BH adjusted p-values < 0.05). Due to statistical correctness, these independent data cannot be pooled together as the experiments and analyses were not performed at the same time (as part of the same TMT-plex, due to the limitations in how many samples could be barcoded and run simultaneously at the time experiments were performed).

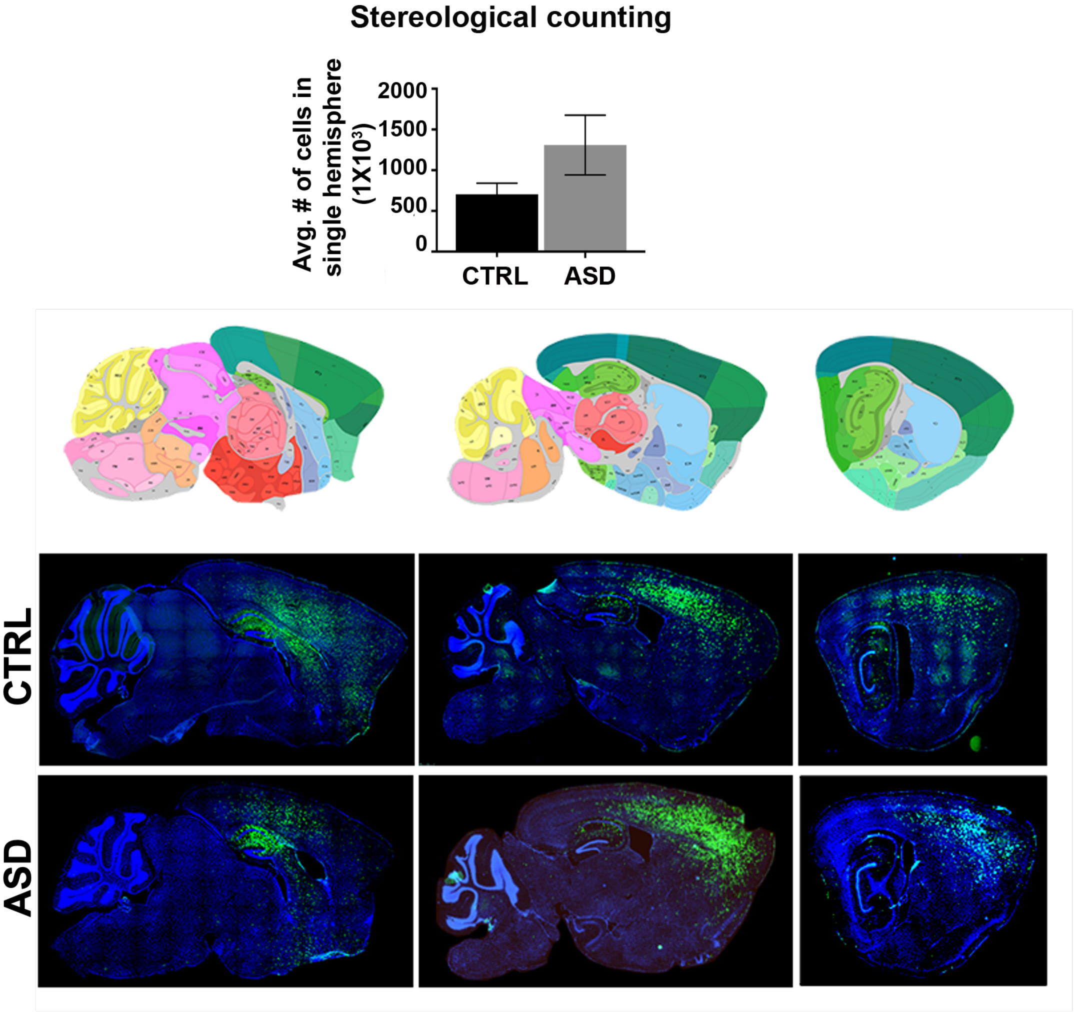

Supplementary Fig. 4: No significant difference in human astrocyte numbers between CTRL and ASD astrocyte chimeric brains, related to Fig. 2.
We dissociated astrocytes from CTRL or ASD cerebral organoids at day 75 and expanded in 2D adherent culture. Astrocytes were labeled with a lentivirus expressing GFP prior to transplantation into *Rag2^{KO}* postnatal mouse brains between P1-3. To assess the average number of astrocytes that survived in host mice, we performed unbiased stereological cell counting on GFP immunostained brain sections. We serially sectioned chimeric brains at P60 along the sagittal plane (30 μm thickness). Every fourth section was used for analysis. Using the optional fractionator method, we estimated the number of surviving cells in one hemisphere from mice transplanted 3 distinct lines for CTRL or ASD. We did not find a significant difference between the numbers of human astrocytes in hemisected CTRL or ASD astrocyte chimeric brains (CTRL: 704,684 ±135,971 GFP+ cells, ASD: 1,309,082 ±367,310 GFP+ cells, unpaired *t* test *p* value = 0.21). CTRL *n* = 3 transplanted brains, 3 distinct lines, ASD *n* = 3 transplanted brains, 3 distinct lines.

Supplementary Fig. 5

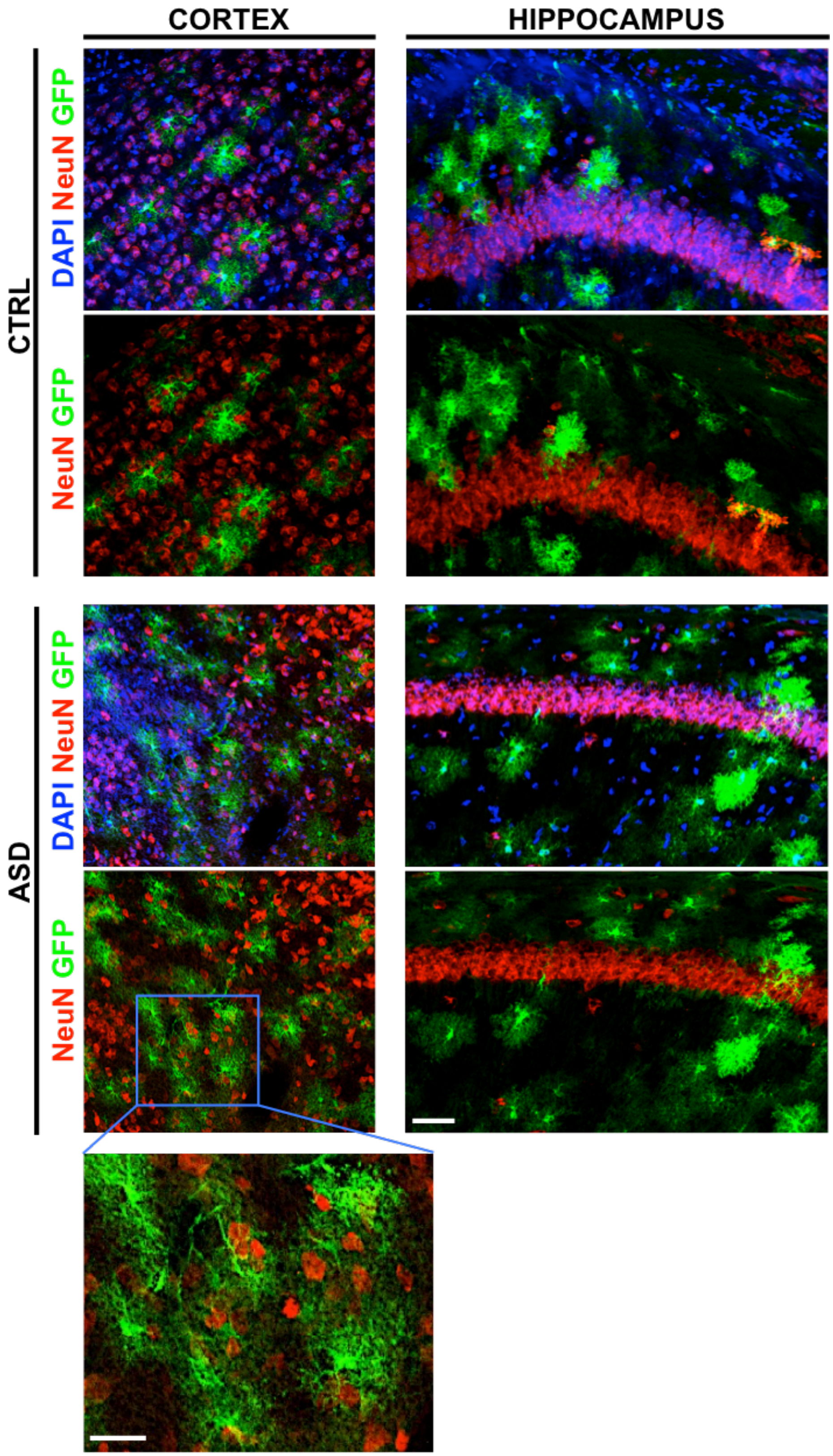

Supplementary Fig. 5: Transplanted human cells do not terminally differentiate to neurons in chimeric brains, related to Fig. 2.

Healthy CTRL or ASD astrocytes were transplanted into the brains of *Rag2*^{KO} mice between postnatal days 1-3 (P1-3). **Fig. 2** shows that more than 90% of the transplanted cells co-localize with known astrocyte markers in adult chimeric brains. Here, we present representative images showing that none of the transplanted GFP+ cells co-localized with the neuronal marker NeuN. We examined over 800 GFP+ cells in 9 chimeric brains (transplanted with 4 distinct iPSC lines/4 CTRL individual and 5 distinct iPSC lines/5 ASD individual; same lines used in behavioral experiments in **Fig. 4**) and found that none of these GFP+ cells expressed nuclear NeuN or exhibited a typical neuronal morphology. In addition to expression known astrocyte markers (**Fig. 2, Supp Fig. 1, and Supp Fig. 3**), GFP+ cells displayed protoplasmic astrocyte morphology in adult chimeric brains. Scale bar = 100 μm for all low magnification panels and 50 μm for the high magnification image at the bottom.

OPEN FIELD

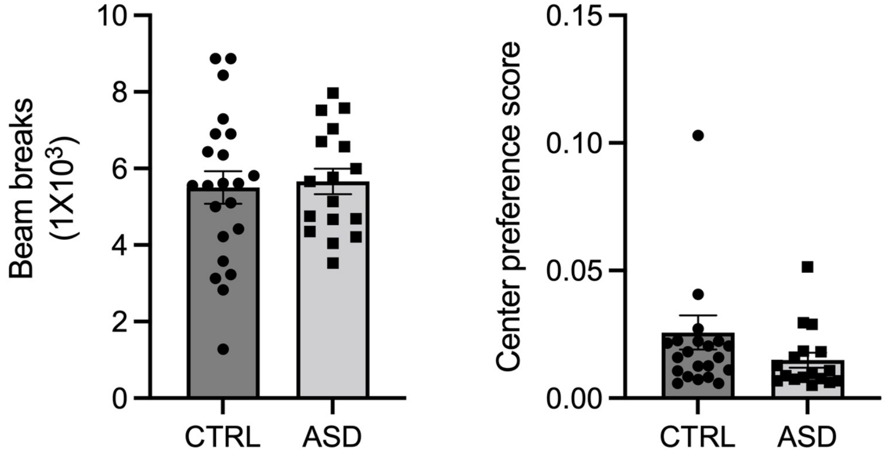

Supplementary Fig. 6: General motor activity is similar between CTRL chimeric and ASD chimeric mice, related to Fig. 4.
We evaluated general locomotor activity in chimeric mice with an automated open field test. ASD chimeric mice did not demonstrate differences in exploratory or general activity relative to CTRL chimeric mice (CTRL 5502 ±423.6 beam breaks, ASD 5659 ±334 beam breaks, unpaired *t* test, *p* value = 0.782). These results suggest that our injection protocol did not affect ambulatory activity and is not a confounding factor in the interpretation of other behavioral results. CTRL *n* = 22 mice (3 distinct lines), ASD *n* = 17 mice (3 distinct lines). A 10 min open field assay was also conducted in locomotor photocells as an additional test, with less time spent exploring the center field of photocells being interpreted as an anxiety-like phenotype. Similar to the outcome of the locomotor activity assay, no evidence of anxiety-like behavior between CTRL (*n* = 22) and ASD (*n* = 22) chimeric mice emerged on this test (*p* value = 0.1363), suggesting that ASD astrocytes did not overtly induced anxiety-like behavior. Data are represented as mean ± SEM.

Supplementary Fig. 7

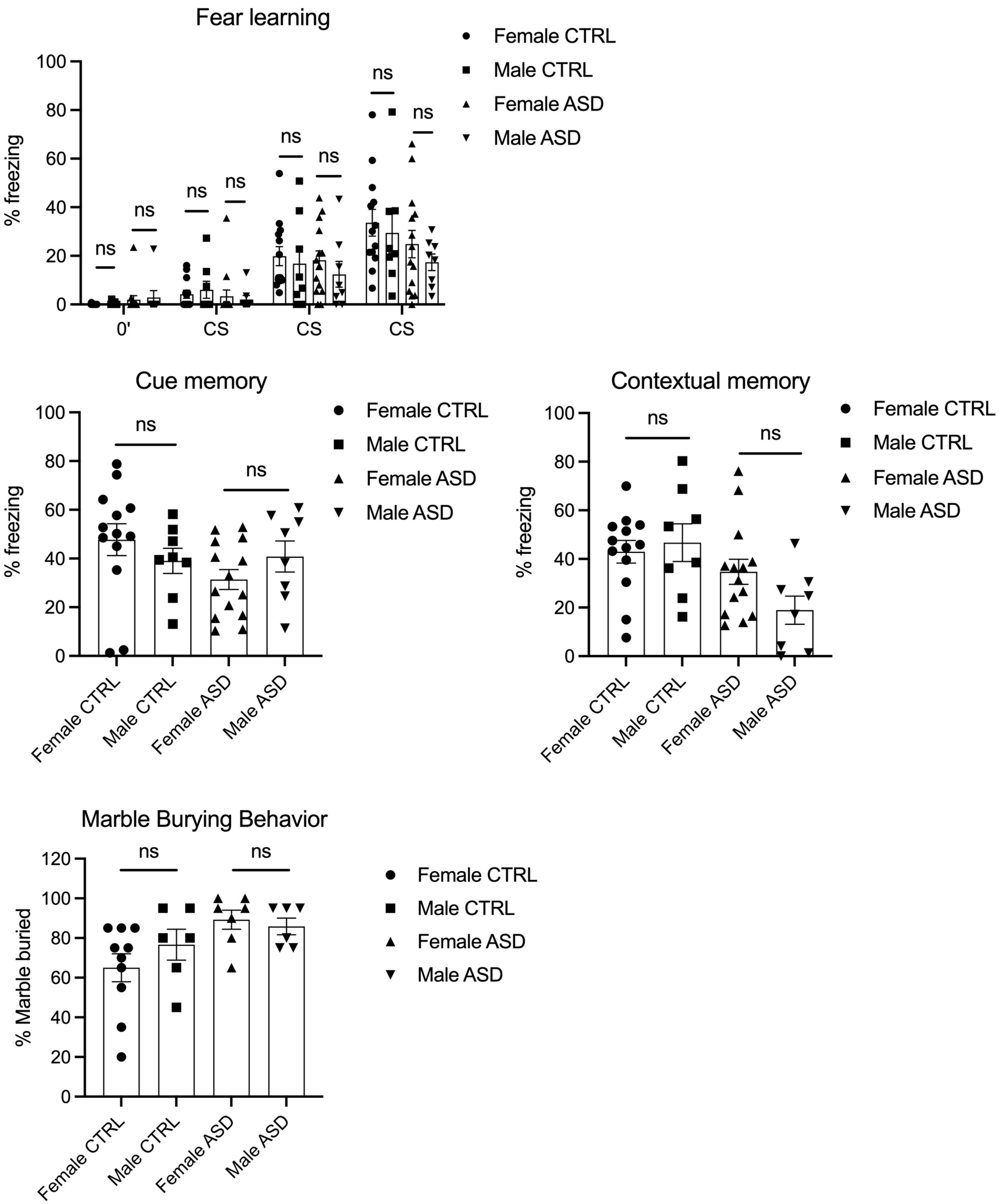

Supplementary Fig. 7: Sex split analyses for fear memory and marble burying performance in CTRL and ASD chimeric mice, related to Fig. 4.

A two-way ANOVA revealed no significant main effect of sex, nor an interaction of sex and ASD transplantation status. The only factor to reach significance was ASD diagnosis. Therefore, sex was not a main effect nor interaction factor that contributed to fear memory and marble burying performance in transplanted chimeric mice.

Fear learning: *p* value Condition (CTRL vs ASD sexes grouped together) 0.0927 N.S.; *p* value Sex 0.4244 N.S.; *p* value Time (% Freezing with increased training) CTRL *p* value < 0.0001 ASD *p* value < 0.0001.

Cue memory: *p* value Condition (CTRL vs ASD) = 0.21956 N.S.; *p* value Sex = 0.9471 N.S.; *p* value Interaction (Condition X Sex) = 0.1297 N.S.

Contextual memory: *p* value Condition (CTRL vs ASD) = 0.0039 *; *p* value Sex = 0.312157 N.S.; *p* value Interaction (Condition X Sex) = 0.104206 N.S.

Marble Burying: *p* value Condition (CTRL vs ASD) = 0.0194 *; *p* value Sex = 0.5450 N.S.; *p* value Interaction (Treatment X Sex) = 0.2695 N.S.

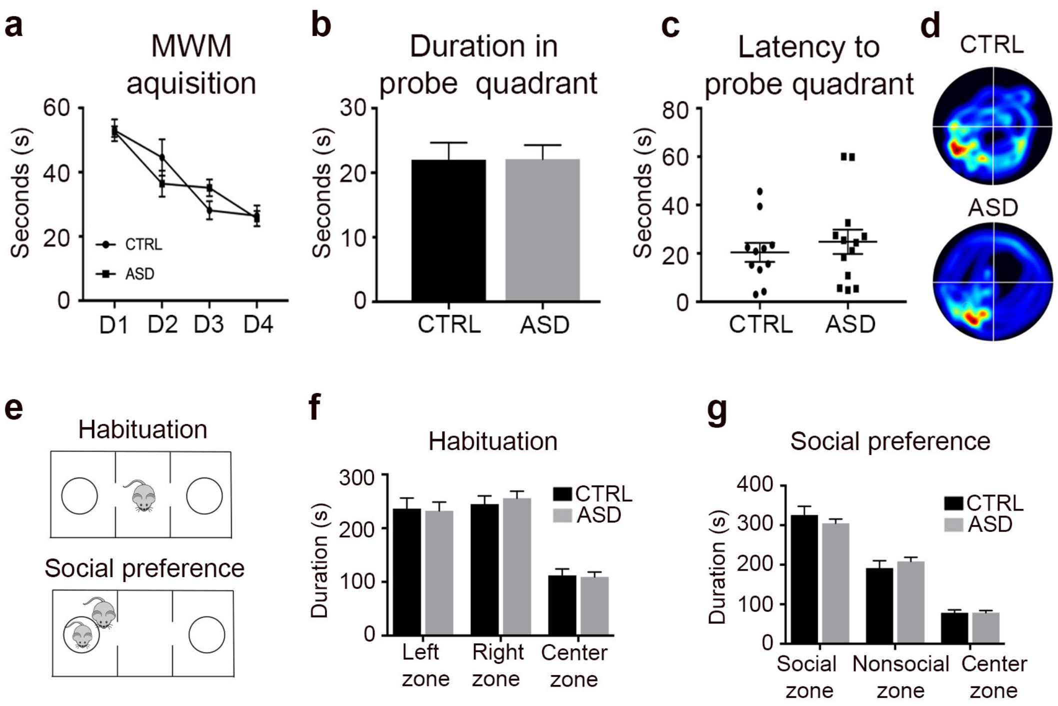

Supplementary Fig. 8: Spatial learning, spatial memory and sociability are not impaired in ASD astrocyte chimeric mice, related to Fig. 4.
a-d, ASD astrocyte chimeric mice showed no deficits in spatial learning or memory when compared to CTRL. To test spatial memory, we used the Morris Water Maze (WMW), where a test mouse relies on distal cues to locate a hidden escape platform submerged in opaque water. Spatial learning is acquired though repeated trials and memory is determined through efficient navigation to an area that formerly housed the escape platform. **a**, no difference between CTRL and ASD mice in the latency to locate the hidden platform during training, which consisted of 4 trials over 4 consecutive days (ANOVA p value > 0.05). **b**,**c,** On the fifth day, the hidden platform was removed and the duration (s) spent in the quadrant that formerly housed the platform was measured. No differences between the latency (s) to reach the correct quadrant were detected between CTRL and ASD (CTRL: 20 ±3.91s and ASD 25 ±5.02s, unpaired t test = 0.51). **d**, Representative heat maps of mouse movements CTRL n = 11 male and female mice, transplanted with 3 distinct lines, ASD n = 13 male and female mice, transplanted with 3 distinct lines.
e-g, To assess sociability, we administered 3-chambered sociability assay in astrocyte chimeric mice (**e**). **f,** During the habituation phase, both CTRL and ASD astrocyte chimeric mice explored the left, right, and center chambers for similar durations suggesting that no bias existed before the insertion of a social stimulus (Left: CTRL

236.35 ±19.76s and ASD 232.41 ±16.13s, Right: 244.82 ±15.24s and ASD 255.77 ±13.18s, Center: CTRL 112.08 ±12.10s and ASD 109.30 ±9.11s, ANOVA with Bonferroni posttests, p value > 0.05). **g**, ASD astrocyte chimeric mice spent a relatively similar amount of time in the social chamber that contained the stimulus mouse compared to CTRL (Social chamber: CTRL 325.74 ±22.14s and ASD 304.71 ±10.77s, Nonsocial chamber: CTRL 191.61 ±18.44s and ASD 208.41 ±10.35s, Center CTRL 78.60 ±7.28s and ASD 78.76 ±5.52s, ANOVA with Bonferroni posttests p value > 0.05). CTRL n = 12 male and female mice, transplanted with 3 distinct lines, ASD n = 22 male and female mice, transplanted with 3 distinct lines. Data are represented as mean ± SEM.

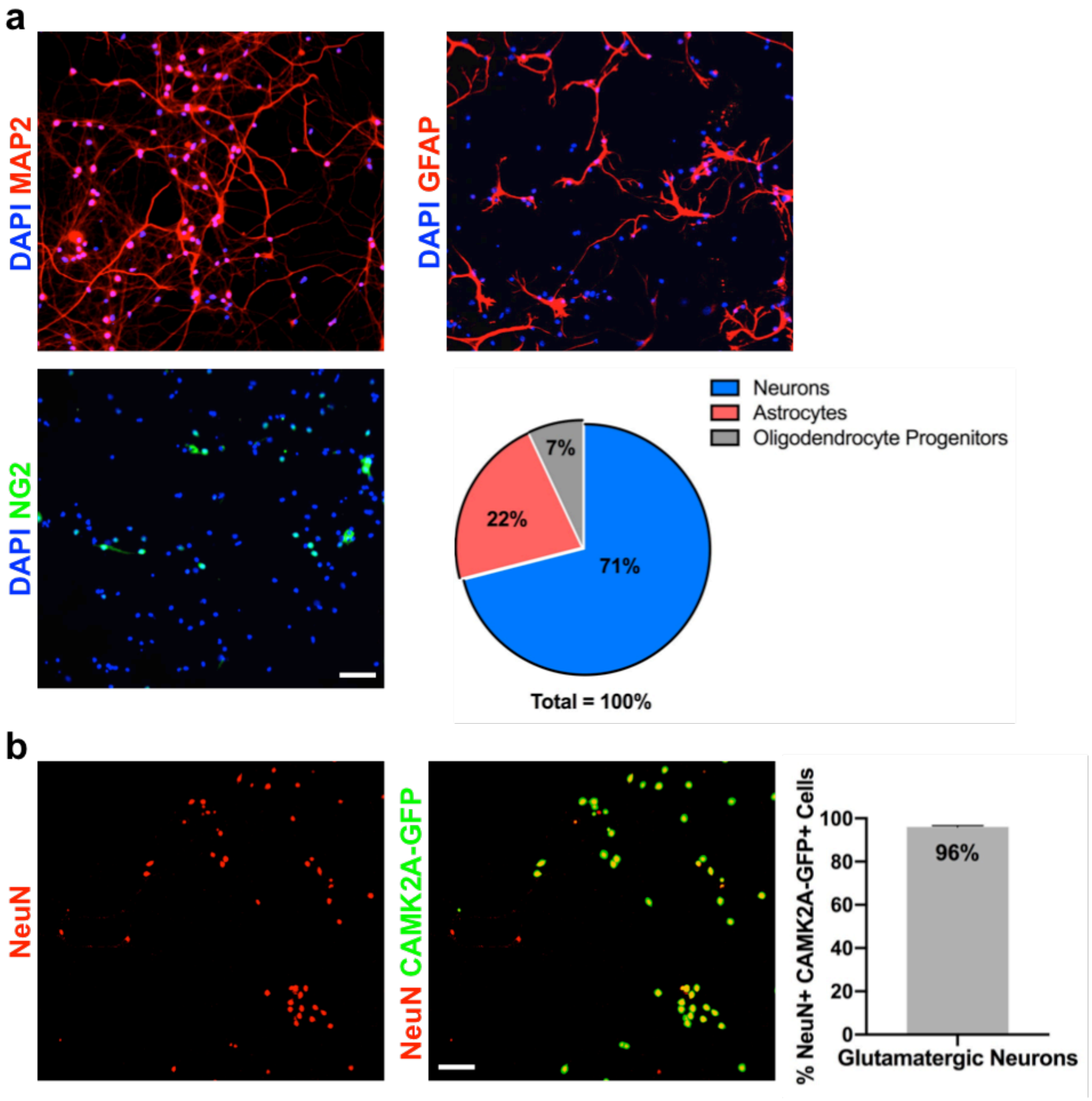

Supplementary Fig. 9: Characterization of mouse hippocampal neuronal cultures, related to Fig. 5. Primary hippocampal neurons were dissociated from the hippocampi of C57BL/6N embryos at E16-18 as previously described[3] and plated $1x10^5$ cells. **a,** At DIV 18, more than 70% of the cultured cells acquired neuron fate, while astrocytes and other glial progenitors represented 22% and 7% of cells, respectively. **b,** Infection of these cultures with a GFP reporter in which nuclear GFP is driven from CAMK2A promoter revealed that more than 95% of the neurons are excitatory glutamatergic neurons.

Supplementary Fig. 10

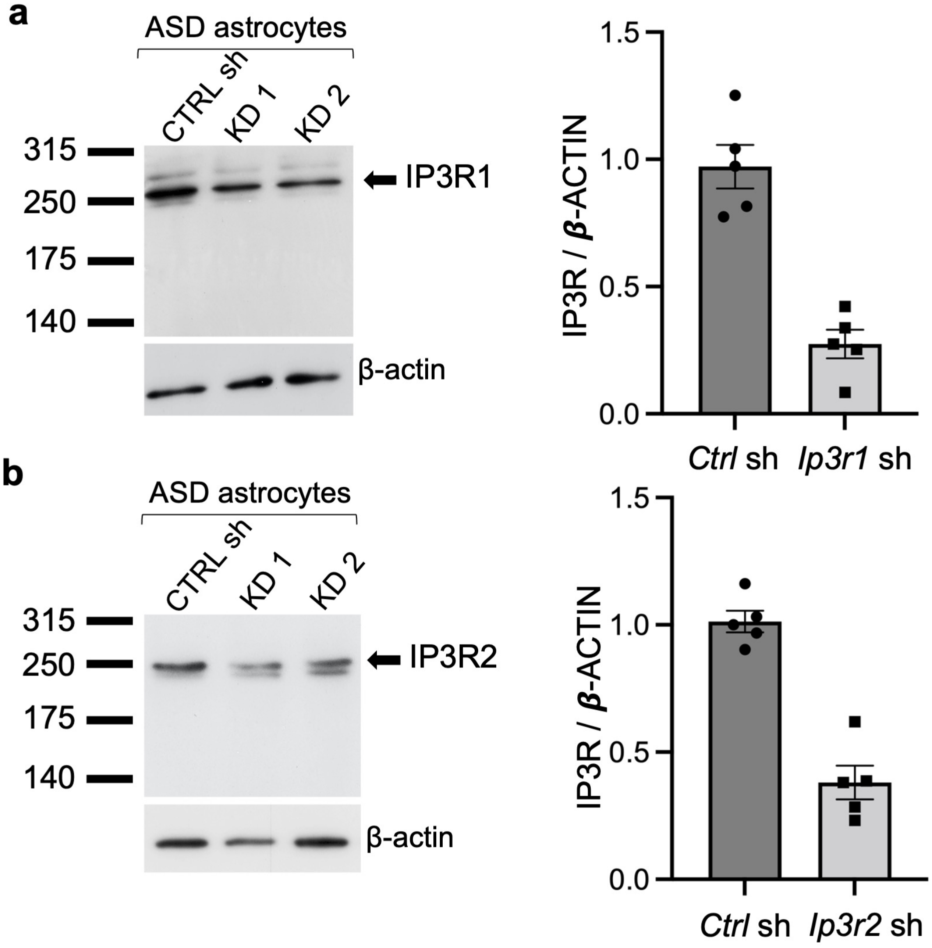

Supplementary Fig. 10: Validation of IP_3R downregulation, related to Fig. 6. To reduce evoked Ca^{2+} increases, we targeted inositol 1,4,5-trisphosphate receptors (IP3R) type 1 and 2 using shRNA based knockdown strategy (KD). We used lentiviral vector carrying a shRNA against *Ip3r1* mRNA (SMARTvector Lentiviral Human ITPR1) (**a**) and lentiviral vector carrying a shRNA against *Ip3r2* mRNA (SMARTvector Lentiviral Human ITPR2) (**b**) from Dharmacon Inc (see Key Resources Table in Methods for purchasing information). To validate the knockdown, we infected astrocytes derived from 5 independent ASD iPSC organoids with these viruses (KD). As a negative control, we infected ASD astrocytes with a non-targeting shRNA lentivirus (Non) (Dharmacon Inc; Key Resources Table in Methods). Cells were lysed 7 days after the infection in RIPA buffer. Western blot analysis showed significant reduction in protein levels of both IP3R1 (**a**) and IP3R2 (**b**) in cells infected with the targeting shRNAs (please see Key Resources Table for antibody information). We also validated the reduction of Ca^{2+} mobilization in these ASD KD astrocytes using a high throughput fluorescent screening assay **(**see **Fig. 6c** for this experiment**).**

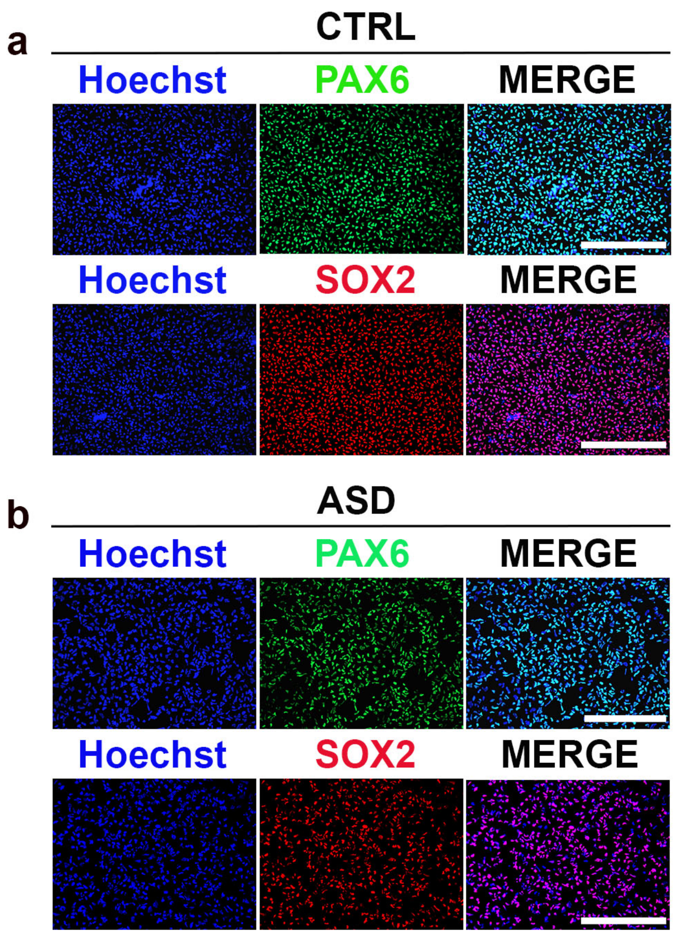

Supplementary Fig. 11: iPSCs derived from ASD patients and CTRL subjects uniformly convert to NPCs, related to Discussions.

a,b, To induce differentiation of iPSCs to forebrain Neural Progenitor Cells (NPCs), an established dual SMAD inhibition protocol was used [4]. Representative images of iPSC-derived NPCs from CTRL subjects (**a**) and ASD patients (**b**) highlighted homogenous conversion with the dual SMAD inhibition protocol as evidenced by expression of PAX6 and SOX2, two early neural progenitor markers. Scale bar = 250 μm.

Supplementary Fig. 12

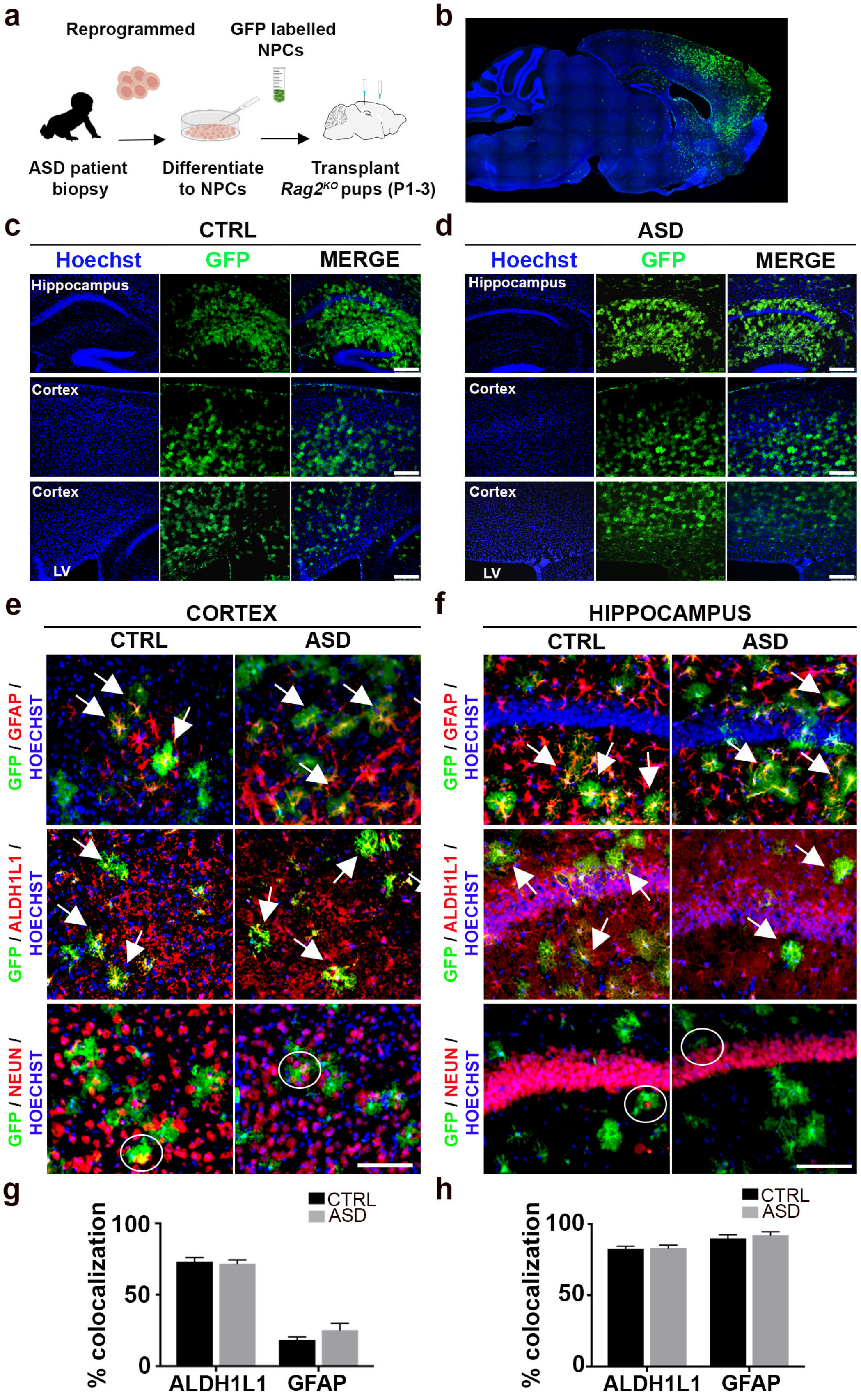

Supplementary Fig. 12: Neural progenitors derived from CTRL and ASD iPSC lines terminally differentiate to astrocytes upon transplantation into the mouse brain, related to Discussions.

Healthy CTRL or ASD neural progenitor cells (NPCs) into the brains of immunocompromised *Rag2*^{KO} mice between postnatal days 1-3 (P1-3). In the healthy developing mouse brain, extensive gliogenesis typically occurs between embryonic day 17 and P17, with peak astrocyte formation occurring between P0 and P2 and oligodendrocyte formation at P14[5]. To induce differentiation of iPSCs to early NPCs, we used an established dual SMAD inhibition protocol[4] (see also **Supp Fig. 11**). Prior to transplantation, human NPCs were labeled with a CAG-GFP lentivirus for detection. **a,** Schematic summarizing the experimental workflow. All immunostainings were performed at P60. **b-d,** GFP+ human NPCs were transplanted into *Rag2*^{KO} mouse brains at P1-3. To visualize the transplantations in the brain, whole brain slices cut on the sagittal plane was immunostained with GFP (**b**, a representative ASD NPC chimeric section). No gross differences in the migration or distribution of human cells in ASD or CTRL NPC chimeric brains was found. **c**,**d,** Representative higher magnification images displayed the extent of human GFP+ cell infiltration throughout the somatosensory cortex and hippocampus in CTRL and ASD transplanted brains. **e**-**h,** GFP+ cells displayed an astrocyte-like morphology in the mature mouse brain. To quantify the number of NPCs that terminally differentiated to astrocytes, chimeric mouse brains were coimmunostained for GFP and either GFAP or ALDH1L1, two well described astrocyte markers. To rule out differentiation to neuronal cell fate, chimeric brains were also coimmunostained for GFP and the neuronal marker, NeuN. Representative coimmunostained images of chimeric brains revealed colocalization of GFP expression (human cells, green) with the expression of two astrocyte markers, GFAP (red, top panel) and ALDH1L1 (red, middle panel) in the cortex (**e**) and hippocampus (**f**). Arrows indicate examples of dual positive cells. Notably, no colocalized expression of GFP (human cells, green) and NeuN (red, bottom panel) was found. See GFP+ processes cradling mouse NeuN positive cell bodies (empty circles). **g**,**h,** More than 70% of GFP expression colocalized with the expression of astrocyte markers both in cortex (**g**) and hippocampus (**h**) (Cortex: ALDH1L1/GFP CTRL 73% ±3% and ASD 72% ±3%, GFAP/GFP CTRL 18% ±2% and ASD 25% ±5%; Hippocampus: ALDH1L1/GFP HP: CTRL 82% ±2% and ASD 83% ±2%, GFAP/GFP CTRL 90% ±3% and ASD 92% ±2%). **Results from these experiments confirm that CTRL and ASD NPCs generated homogenous, high-density transplantations in host brains and primarily differentiated into astrocytes upon maturation in the living brain.**

Scale bar = 250 μm for c-f. Immunostaining quantifications: CTRL and ASD n = 12 per group (3 brains transplanted with distinct lines per group and 4 slices per brain). Data are represented as mean ± SEM.

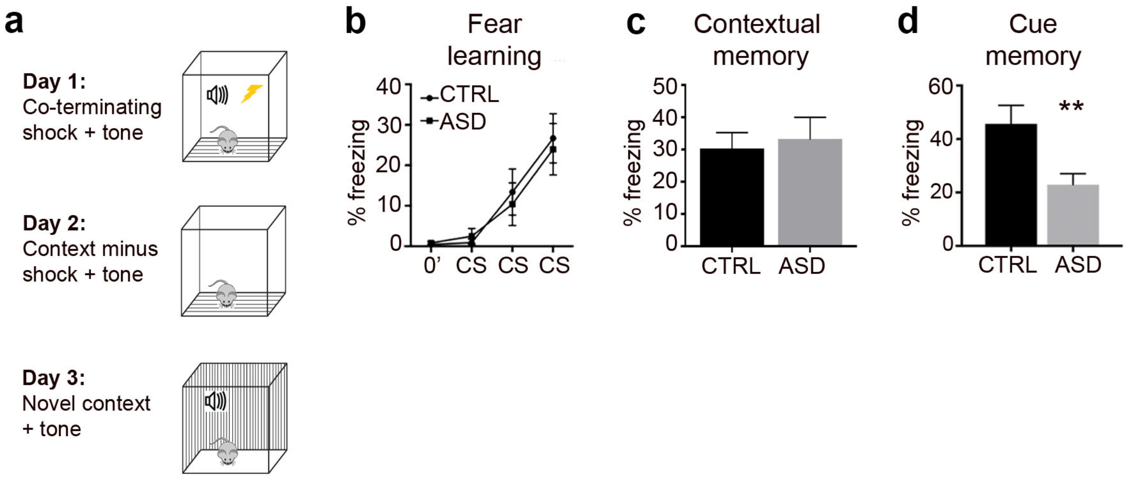

Supplementary Fig. 13: ASD NPC chimeric mice display impaired associative memory but not learning deficits, related to Discussions.

CTRL or ASD neural progenitor cells (NPCs) were engrafted into the brains of $Rag2^{KO}$ mice between postnatal days 1-3 (P1-3). Gliogenesis typically occurs between embryonic day 17 and P17, with peak astrocyte formation occurring between P0 and P2[5]. Greater than 70 percent of transplanted human CTRL or ASD NPCs acquired an astrocytic fate *in vivo* (**Supp Fig. 12**).

a-d, NPC chimeric mice were tested for deficits in learning and memory with a classical fear conditioning protocol. In this paradigm, freezing behavior represented a species-specific response to fear and was defined as the absence of movement except for respiration. **a,** Schematic summarizing classical fear conditioning paradigm. On day 1, mice learned to associate an audible tone (30 sec duration, 70dB) with a co-terminating foot shock (1 sec duration, 0.7mA). Testing days 2 and 3 measured freezing behavior in response to exposure to the training context or the audible cue in a novel context, respectively. Freezing behavior during these testing trials provided a quantifiable measure of associative memory. **b,** Both CTRL and ASD NPC chimeric mice learned the association as evidenced by increased freezing behavior in response to each successive tone-shock pairing (ANOVA with Bonferroni posttests *p* value = 0.0001). We found no significant differences in the rate of learning between CTRL and ASD chimeric mice (ANOVA with Bonferroni posttests *p* value > 0.05).

c,d, Transplantation of ASD NPCs did not influence contextual associative learning (CTRL 30.33 ±4.91%, ASD 33.38 ±6.69%, unpaired *t* test, *p* value = 0.73) (**c**). However, ASD NPC chimeric mice displayed lower freezing levels than CTRL during the cued memory trial indicating impaired associative memory (CTRL 45.68 ±6.89%, ASD 22.95 ±4.07%, unpaired *t* test, *p* value = 0.007) (**d**). CTRL n = 12 mice, transplanted with 3 distinct lines, ASD n = 14 mice, transplanted with 4 distinct lines. Data are represented as mean ± SEM.

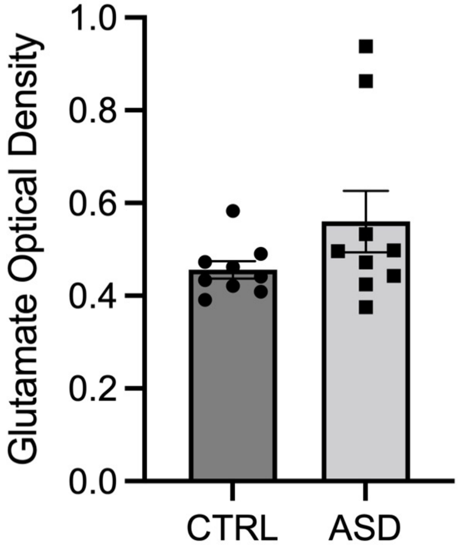

Supplementary Fig. 14: Glutamate levels are not significantly different between CTRL and ASD astrocytes, related to Discussions. We measured glutamate levels in CTRL and ASD organoid-derived astrocyte supernatant (Passage 8) using a glutamate assay kit (ab83389) (see Methods section). Supernatant was collected 96 hours after passaging and showed no difference in glutamate levels between CTRL and ASD astrocytes (*p* value: 0.16; *n* = 9 distinct lines for ASD and *n* = 9 distinct lines for CTRL). Data are represented as mean ± SEM.

Supplementary Video 1, Related to Fig. 1: ASD astrocytes respond to stimulation with more intense Ca^{2+} transients compared to CTRL astrocytes.
To acquire Ca^{2+} signals, we first loaded astrocytes with a Ca^{2+} indicator dye (Fluo-4-am, 1μM) and incubated for 30 min at 37°C. The dye was completely washed away and cells permitted to equilibrate at 37°C for 30 min. We used Olympus RS equipped with a scanning galvanometer and a Spectra-Physics Mai Tai DeepSee laser for the excitation of GFP. We recorded fluorescence through gallium arsenide phosphide (GaAsP) detectors using the Fluoview acquisition software (Olympus) with a green light emission bandpass filter (Semrock).

Supplementary Video 2, Related to Fig. 5: MEA recordings from DIV14 human astrocyte and mouse neuron co-cultures and mouse neurons only control culture. Each column represented an astrocyte derived from a unique subject (CTRL n=3 and ASD n=4). Heat map of real-time firing visually showed that CTRL co-cultures had more spontaneous network activity than ASD co-cultures.

Supplementary Video 3, Related to Fig. 7: MEA recordings from DIV14 human astrocyte and mouse neuron co-cultures. Human astrocytes were transduced control (non-targeting, Non) or ITPR knockdown (KD) shRNA and then added to culture with mouse primary neurons. Shown are CTRL Non co-cultures with astrocytes derived from two unique patients. ASD co-cultures were grouped by unique patient. KD treatment in ASD co-cultures increased neuronal network activity so that there was no significant difference when compared to the activity of CTRL Non co-cultures.

Methods

Materials availability
All unique/stable reagents generated in this study are available from the Corresponding Author with a completed Materials Transfer Agreement.

Data and code availability
The datasets supporting the current study have not been deposited in a public repository because data is embargoed until publication acceptance at which point the dataset will be publicly available from the proteomics databases PRIDE and/or PepditeAtlas, as consistent with editorial standards.

Key resources table

**Cell Culture**		
**Reagent**	**Source**	**Identifier**
**Astrocyte culture**		
TrypLE + phenol red	Gibco	12605028
DMEM, high glucose, GlutaMAX™ Supplement	Thermofisher	10566024
Penicillin-Streptomycin (10,000 U/mL)	Thermofisher	15140-122
Fetal Bovine Serum	Corning	35-010-CV
Dimethyl sulfoxide ≥99.5% (GC), plant cell culture tested	Sigma	D4540-500ML
Deoxyribonuclease (DNase) I from bovine pancreas	Sigma	dn25
**Stem cell culture**		
Vitronectin (VTN-N) Recombinant Human Protein, Truncated	Thermofisher	A14700
Essential 8 Medium	Thermofisher	A1517001
Penicillin-Streptomycin (10,000 U/mL)	Thermofisher	15140-122
Rock Inhibitor (compound Y-27632)	StemCell Technology	72304
Accutase	Sigma	A6964-100ML
Dulbecco's Phosphate-Buffered Saline, 1X without calcium and magnesium	VWR	45000-434
EDTA, 0.5 M, pH 8.0, Molecular Biology Grade	Sigma	324506
**Neural progenitor culture**		
Essential 6 medium	Thermofisher	A15169.01
Penicillin-Streptomycin (10,000 U/mL)	Thermofisher	15140-122
SB-431542	Tocris	1614
LDN 193189 dihydrochloride	Tocris	6053
**Organoid culture**		
DMEM/F-12, GlutaMAX™ medium	Thermofisher	10565042

KnockOut Serum Replacement	Thermofisher	10828028
Fetal Bovine Serum	Corning	35-010-CV
GlutaMAX™ Supplement	Corning	35-010-CV
MEM-NEAA	Sigma	M7145-100ML
2-Mercaptoethanol	VWR	200000-620
Penicillin-Streptomycin (10,000 U/mL)	Thermofisher	15140-122
Y-27632 compound (ROCK inhibitor)	StemCell Technology	72304
bFGF 4ng/ml	Peprotech	100-18B
N2 Supplement	Thermofisher	17502048
MEM-NEAA	Sigma	M7145-100ML
Penicillin-Streptomycin (10,000 U/mL)	Thermofisher	15140-122
Heparin sodium salt from porcine intestinal mucosa	Sigma Aldrich	H3149-10KU
Matrigel™ Matrix	VWR	47743-715
Neurobasal® Medium, minus phenol red	Thermofisher	12348017
B-27 Supplement (50X), minus vitamin A	Thermofisher	12587010
B-27 Supplement (50X), plus vitamin A	Thermofisher	17504001
Insulin	Sigma Aldrich	I9278-5ML
Fungizone Amphotericin B solubilized	Sigma	A9528-100MG
**Equipment**		
Ultra-Low Attachment Multiwell Plates, Sterile, 24 well	VWR	29443-032
Ultra-Low Attachment Multiwell Plates, Sterile, 96 well	VWR	29443-034
Nunc™ Cell Culture Treated Flasks with Solid Caps 25 cm2	VWR	15708-097
Nunc™ Cell Culture Treated Flasks with Solid Caps 80 cm2	VWR	15708-106
VWR® Tissue Culture Dish, Non-Treated, Sterilized, Non-Pyrogenic 6.0 cm	VWR	10861-588
Nunclon™ Delta MultiDishes, Sterile, 6 well plate	VWR	73520-906

IMMUNOHISTOCHEMISTRY

Reagent	Source	Identifier
Hydromount	National Diagnostics	HS-106
PBS (10X), pH 7.4	Thermofisher	70011069
PFA 20% Solution	EMS	100496-494
Tissue-Tek O.C.T. Compound, Electron Microscopy Sciences	VWR	102094-104
Triton® X-100 Surfactant	Millipore Sigma	TX1568-1
Goat Serum (NGS)	Sigma Aldrich	G9023-10ML
**Antibodies**		
GFP Tag Polyclonal Antibody (1:1000)	Thermofisher	A10262
GFAP (1:1000)	Agilent	Z0334
ALDH1L1 antibody (1:300)	Abcam	ab190298

NeuN (1:500)	Millipore sigma	MAB377
Beta III Tubulin Antibody (1:1000)	EMD Millipore	AB9354
Aquaporin 4 Antibody, CT (1:500)	Millipore Sigma	AB3594-50UL
S100Beta mouse monoclonal (1:500)	Sigma Aldrich	S2532
Vimentin (R28) Antibody (1:500)	Cell Signaling	3932
huGFAP (1:1000 + amplification)	Biolegend	SMI-21
IP3 Receptor 1 Polyclonal Antibody	Thermofisher	PA1-901
IP3 Receptor 2 Polyclonal Antibody	Thermofisher	PA1-904
Goat anti-Mouse IgG (H+L) Secondary Antibody, Alexa Fluor® 488 conjugate	Thermofisher	A-11001
Goat anti-Rabbit IgG (H+L) Secondary Antibody, Alexa Fluor® 488 conjugate	Thermofisher	A-11008
Goat anti-Mouse IgG (H+L) Secondary Antibody, Alexa Fluor® 546 conjugate	Thermofisher	A-11003
Goat anti-Rabbit IgG (H+L) Secondary Antibody, Alexa Fluor® 546 conjugate	Thermofisher	A-11035
Goat anti-Chicken IgY (H+L) Secondary Antibody, Alexa Fluor® 546 conjugate	Thermofisher	A-11040

Recombinant proteins, peptides, and small molecules

Reagent	Source	Identifier
Thapsigargin	Tocris	1138
Norepinephrine	Sigma	A9512
Endothelin 1	Sigma	694105
ATP Solution	ThermoFisher	R0441
DHPG	Tocris	0342
Forskolin	Sigma	F6886
Fluo-4-AM	ThermoFisher	F14201
Fluo-8-AM calcium flux assay kit	Abcam	ab112129
Alexa Fluor™ 488 Phalloidin	ThermoFisher	A12379
Glutamate Assay Kit	Abcam	ab83389

VIRAL VECTORS

Reagent	Source	Identifier
SMARTvector Nontargeting Control shRNA hCMV-RFP UHT, 2x10^9 TU/mL	Dharmacon Inc	VSC7350
SMARTvector Lentiviral Human ITPR1 hCMV-TurboRFP shRNA UHT, 2x10^9 TU/mL	Dharmacon Inc	V3SH7602-225253796
SMARTvector Lentiviral Human ITPR2 hCMV-TurboRFP shRNA UHT, 2x10^9 TU/mL	Dharmacon Inc	V3SH7602-227028601
CAG-GFP lentivirus	Addgene	16664
αCaMKII-GFP AVV virus	Addgene	50469-AAV5
gfaABC1D-cyto-GCaMP6f	Addgene	52925-AAV5

pENN.AAV.CamKII.HI.GFP-Cre.	Addgene	105551-AAV9

MICE		
Strain	Source	Stock No.
C57/6N pregnant females	Charles River	027
RAG2^{KO} mice	Jackson Labs	008449

iPSC LINES		
Name	Source	Identifier
CW20081FF1	CIRM	CW20081
CW2014DD1	CIRM	CW20142
CW60115FF1	CIRM	CW60115
CW20083AA1	CIRM	CW20083
CW20008FF1	CIRM	CW20008
MH0148698	NIMH	MH0148698
MH0148713	NIMH	MH0148713
CW20026	CIRM	CW20026
CW20044	CIRM	CW20044
MH0174677	NIMH	MH0174677
MH0159021	NIMH	MH0159021
MH0159020	NIMH	MH0159020
MH0174686	NIMH	MH0174686
MH0159019	NIMH	MH0159019
MH0174679	NIMH	MH0174679
MH0174681	NIMH	MH0174681
GM23279	Coriell	GM23279
GM25256	Coriell	GM25256

IMAGING EQUIPMENT	
Instrument	Manufacturer
Epifluorescent microscope	Leica
Confocal fluorescent microscope	Zeiss
2-Photon live cell imaging microscope	Olympus
Microplate reader for Ca^{2+} flux assays	Hammmatsu FDSS 6000

SOFTWARE	
Name	Website
Ethovision	www.noldus.com
MATLAB	www.mathworks.com
Graphpad PRIZM	www.graphpad.com
ImageJ	www.imagej.nih.gov

Mice. Animal experiments were conducted in accordance with the ethical guidelines of the National Institutes of Health and approved by the Institutional Animal Care and Use Committee at Weill Cornell Medicine. All animals were group housed with littermates and permitted free access to food and water. Experimental results compare *Rag2^{KO}* immunocompromised mice transplanted with cells derived from ASD iPSC lines or CTRL iPSC lines. Neuronal cell cultures were dissociated from E18 pregnant C57/6N females purchased from Charles River.

Induced pluripotent stem cells (iPSCs). iPSC lines were purchased from NIH and CIRM repositories (Supplementary Table 1, see also Key Resources table in Supplementary Methods). Two CTRL lines were purchased from Coriell Institute. Each repository characterized and validated cells as pluripotent and performed karyotyping to ensure the genomic integrity of each reprogrammed line. ASD lines MH0148698 and MH0148713 from an NIMH collection (Study 116) were genetically characterized and published[6]. Please see Supplementary Tables 1-3 for clinical as well as genetic information. Key resources table includes information for purchasing. Supplementary Table 4 provides a list of experiments and specific lines used for each experiment. Our decision to use iPSC lines derived from non-family CTRLs was influenced by studies that show neuropsychiatric disorders cluster in families[7]. First-degree relatives of individuals with ASD display behavioral or cognitive features associated with autism that does not meet diagnostic criteria, such as social or language dysfunction[8-14]. Similarly, autistic-like social impairment is increased in unaffected parents and children of people diagnosed with ASD[15,16]. Therefore, using a CTRL group with no history of psychological, genetic or other disorders would allow us to detect subtle changes in our ASD group as well as determine whether there are common alterations in astrocyte biology and function among the disparate genetic origins of ASD. In fact, multiple studies to date have presented robust cellular phenotypes in iPSC-derived cells for nonsyndromic disorders, which are also characterized by a high degree of genetic heterogeneity, when non-family controls were used[17-22]. All ASD iPSC lines were derived from male (Supp table 1). Thus, to keep consistence as sex differences are implicated in ASD, gender also played a major criterion in our selection of CTRL lines in addition to availability and extensive characterization by the NIMH repository. Typically, exclusively male CTRL lines or male + one female CTRL line were used in the experiments. Only in genetic characterization experiments (whole exome sequencing) and astrocyte reactivity assessments were both female CTRL lines included along with all other male lines. In general, a prevailing consensus is that sex is unlikely to be a significant factor in these sorts of iPSC studies given a lack of sex-hormone expression in studied cell types (and this is consistent with our own observations in-house). Supplementary Table 4 summarizes the specific iPSC lines that were used in each experiment. In Proteomics studies, 9 male ASD and 7 male + 1 female CTRL lines were used. Proteomics analysis suggested that Ca^{2+} signaling is altered in ASD astrocytes.

Only male CTRL lines were used in *in vivo* and *in vitro* Ca^{2+} experiments (**Fig. 1 and Fig. 3**). Similarly, in experiments where we measured neuronal network activity as well as rescued the network activity phenotype by modulating Ca^{2+} fluctuations (**Fig. 7**), only male CTRL lines were used. In behavioral and LTP experiments, only 1 female CTRL line, together with 4 male CTRL lines, was used. Thus, taking into account the altered Ca^{2+} activity phenotype in ASD astrocytes underlying subsequent alterations in neurons, our mechanistic conclusions derive from the use of only male CTRL and male ASD lines.

Whole Exome Sequencing (WES). DNA from astrocytes was isolated and subjected to NovaSeq6000 to generate 2x151bp paired-end reads at an average depth of 30X. For genetic analysis, whole exome sequencing analysis was performed in all control and ASD lines used here. The detected variants (both SNV/indel and CNV) that overlapped known variants in ClinVar that have been implicated in autism with a clinical significance of "Pathogenic" or "Likely Pathogenic" were screened in ASD and control group resulting in a screening panel of 2367 SNVs/indels and 385 CNVs. No relevant hits for SNVs/indels in either ASD or control group were found with these established autism variants. 25 CNVs in ASD and 31 CNVs in controls that had a 50% reciprocal overlap with ClinVar CNVs, all of which were duplications (aside from one deletion) that overlapped non-critical regions of "Pathogenic" or "Likely Pathogenic" CNVs were detected. We also compiled a screening panel on the gene level comprised of 322 ASD-related genes obtained from SFARI Gene database (from the high confidence category) as well as human genes from the MGI database that have been linked with autism. Across these 322 genes, 33 rare and potentially impactful variants in ASD samples and 37 variants in control samples were identified. All of these variants, with the exception of 2 in-frame insertions, are missense variants with a 'moderate' predicted impact. None of these variants harbor a "Pathogenic" or "Likely Pathogenic" classification by ClinVar at the variant level. Supplementary table 2 displays the counts of hits for LGD SNVs and LGD CNVs. Supplementary table 3 displays variants in ASD and controls and which individual carries them.

iPSC cultures. All iPSC lines were thawed and directly plated on vitronectin (1μg/cm^2) coated 6-well tissue culture treated plates. ROCK inhibitor (10 μM) was added to culture media for the first 24 h after thawing. Otherwise, iPSC cultures were maintained in E8 base media plus E8 supplement, and 1X penicillin/streptomycin antiobiotic and incubated at 37°C with 5% CO_2 humidified air. Media changes occurred 6 days a week. We surveyed wells daily and passaged with 0.5 mM EDTA once colonies reached 70-80% confluency. Optimal iPSC lines formed spherical colonies with slightly raised edges. If spontaneous and excessive differentiation occurred, undifferentiated colonies were manually isolated under a dissection scope with sterile pipette tips and transferred

to newly vitronectin coated 6- or 12-well culture treated plates. No ROCK inhibitor was added when manually transferred.

Organoid development. iPSCs were cultured into three-dimensional embryoid bodies, induced neural fate specification, and allowed free floating tissue to expand for 75 days in 10 cm sterile tissue culture dishes. During this expansion phase, organoids were maintained in media (DMEM-F12, Neurobasal medium, N2 supplement, B27 supplement + vitamin A, insulin, GlutaMAX supplement, 2-mercaptoethanol, MEM-NEAA, penicillin-streptomycin, and fungizone) and agitated at 60 RPM on an orbital shaker to ensure nutrient diffusion. Because this organoid protocol does not use excessive patterning factors or small molecules, it likely preserves disease-specific signatures unique to ASD astrocytes. Cell fate specification is temporally regulated in cortical development and is defined by the sequential appearance of neurons followed by glia[5]. In this system, ASD astrocytes develop in the presence of genetically matched ASD neurons. This is important because astrocytes require early interaction with neurons to induce expression of critical receptors and trigger temporally regulated activation patterns[23].

Astrocyte expansion. Organoids (DIV75) were enzymatically dissociated in a 0.1% TRYP-LE solution for 15 min and washed 2X with astrocyte selection media (DMEM supplemented with 4.5 g/L of D-glucose, 2mM GlutaMAX™, 2% fetal bovine serum and antibiotic and fungicide). Gentle trituration with a fire polished glass pipette was used to obtain a single cell suspension. This suspension was plated on tissue culture treated 6-well plates at a density of 1.5 million cells per well. Astrocyte selection media supplemented with 10 µM ROCK inhibitor was added to the cells the first 24 h after dissociation. Astrocyte expansion occurred in 2D adherent culture conditions with D-glucose, GlutaMAX™, 2% fetal bovine serum and antibiotic and fungicide. Previous studies have shown that high levels of serum (>10%) result in reactive phenotypes that are not representative of astrocytes *in vivo*[24,25]. In our hands, minimal serum exposure enhanced astrocyte survival and proliferation and permitted large-scale astrocyte production without causing reactivity. Neurons co-cultured with CTRL astrocytes derived in this way survived and retained neuronal network activity that was similar to neurons without human astrocytes (refer to **Fig. 5**).

Proteomics. Proteins were extracted from cell pellets and quantified with the Bradford assay. Samples were enzymatically digested, labeled with tandem mass tags (TMT), and then combined. The combined TMT-labeled peptides were desalted and subject to LC/MS analysis. MS raw data was searched against Uniprot human database using MaxQuant and Perseus Student's T-test was performed and p values were adjusted with Benjamini-Hochberg (BH) Correction to avoid false positives. All statistical and bioinformatics analyses were performed using the freely available software Perseus (as

part of the MaxQuant environment), the R framework, or EdgeR framework. Proteins identified only by site modification or found in the decoy reverse database were not considered for data analysis. The intensities of proteins were used calculate the ratio and the p-value using EdgeR. Differentially expressed proteins were identified as follows: ratio ±1.5(log2), p-value <0.05. Pathway enrichment analysis for categorical data was performed based on a Fisher's exact test with a Benjamini–Hochberg FDR threshold of 0.02. Coverage of calcium-related gene ontology enrichment (molecular function). GOMF, KEGG and Uniprot Keyword annotations were used for enrichment analysis using DAVID informatic platforms and Reactome and required a minimum category size of at least three proteins. Percentages of coverage in the indicated MF GO categories are shown. KEGG pathway analysis, were applied to the data, identifying members of major biological processes including muscle contraction and adhesion related (focal adhesion, tight junction) pathways, with percentages of pathway coverage in the indicated KEGG categories shown. Data were subsequently also plugged into Qiagen's ingenuity pathway analysis (IPA) to predict differences in functional protein signaling networks. A 75% confidence interval was used for prediction analysis for two independent proteomic experiments. Next, IPA software compared the two independent results and determined calcium signaling as the most significantly altered pathway (95% confidence interval, BH corrected *p* value).

Transplantations. Astrocyte or neural progenitor human chimeras were prepared as described[26,27] with minor modifications. Prior to transplantation, astrocytes were transduced with a CAG-GFP lentivirus to distinguish human derived cells from endogenous mouse cells *in vivo*[28]. Cells were resuspended in the DPBS at a concentration of $5x10^5$ cells/µl and maintained on ice. Newborn *Rag2*^{KO} pups (P1-3) were cryoanesthetized and $2.5x10^5$ human derived cells were injected in equal amounts (0.5 µL) into the forebrain at four locations ($8\text{-}10x10^5$ in total for each brain). Bilateral injections were made immediately anterior and posterior to bregma. Injections were made by hand using a Hamilton syringe (30 gauge) and inserted gently through the skin and skull. Pups were monitored twice a week until weaning.

Stereology*.* Stereo Investigator Stereological Software (MBF Bioscience, Williston, VT) was used to estimate the total number of GFP+ human astrocytes in hemispheres of chimeric mice. Chimeric mice underwent transcardial perfusion with 4% PFA at P60 and brains were cryosectioned along the sagittal plane (30μm thick). Every 4th slice was taken for analysis. Slices underwent immunostaining to enhance GFP signal. Sections were traced under low magnification (4X) and counted under high magnification (20X) using the optical fractionator method. Briefly, the software would systematically and randomly choose counting frames within the brain section. Then, it would draw an 80μm × 80μm square around the counting frame. Cells were counted if they were found within

that box and only if they did not come into contact with two borders of square frame. Upon completion of counting, we applied the numerical formula supplied by MBF bioscience to extrapolate the total cell number within each brain hemisphere. To calculate estimates, we multiplied the total number of cells counted by the total volume of the brain slice (mm^2) divided by the volume of counting square (mm^2).

Behavioral testing. Aged matched cohorts were housed in temperature-controlled rooms with a 12-h light/dark cycle. Cages were changed weekly; care was taken not to test animals on the same day as cage change. To further minimize confounding results due to stress, mice were handled for 3 consecutive days prior to the start of testing. When possible, the experimenter was blinded to engraftment group. For all behavioral assays, mice were habituated to the testing room for a minimum of 30 min, and testing was performed at the same time of day except where noted. Animals were 3-5 months of age at the time of testing.

Open Field. We tested general ambulatory activity and exploratory behavior of chimeric mice with an open field test (San Diego Instruments). Four boxes, each measuring 50cm × 50cm, allowed for the simultaneous testing of up to four mice. Each box contained a 16 × 16 photobeam configuration that reported beam breaks in x, y, and z planes. The associated PAS software accurately recorded all beam interruptions in real time to report central and peripheral localized activity, ambulatory movements, fine movements, and rearing.

Sociability. To test sociability in mice, a 3 chambered social approach assay (with slight modifications) was used[29]. Testing occurred in a 3-chambered apparatus where animals were free to access to all chambers during testing. The test was divided into two phases: habituation and social preference. During the habituation phase test animals were placed in center of apparatus box that contained two identical wire mesh cylinders at each end chamber. Animals were monitored for 10 min during each phase. Next, a stimulus mouse was placed in one of the wire mesh cylinders. Because testing occurred over multiple days, placement of the social cylinder was counterbalanced throughout testing to eliminate any bias toward any chamber in the apparatus. The time spent in the social or non-social chamber was recorded over a 10-min testing period as well as the time spent interacting with the cylinder that contained the social stimulus. Automated analysis was performed with Noldus Ethovision XT software.

Fear learning and memory. Context and cue dependent fear conditioning is an associative learning task where mice learn to associate a neutral conditioned stimulus (CS; audible tone, 70 dB, 30 sec duration) with an aversive unconditioned stimulus (US; mild electrical foot shock, 0.7 mA, 1 sec duration) and display a conditioned response (CR; freezing behavior)[30]. The CR or freezing behavior can be used as an index of learning and memory. After repeated pairings of CS and US, the animal learns to fear both the tone (cue-dependent memory) and training context (context dependent

memory). Chimeric mice learned to associate a neutral conditioned stimulus (CS; audible tone) with an aversive unconditioned stimulus (US; mild electrical foot shock) during a 5-min training session. On day one, mice were exposed to a 5-min conditioning trial that involved three co-terminating tone shock pairings. On day two, mice were returned to the conditioning context for 5 min, with % freezing measured for the first 2 min of the test session. This measured the association of the memory to the context. On day three, mice were placed in a novel context and presented only the audible tone. Freezing behavior to the first tone presentation was recorded and represents cue-associated memory. We reported average freezing % during the first tone presentation. On all testing days, % freezing behavior was a readout for fear memory. Freezing percentages were determined by automated software from Med Associates Inc.
Spatial learning and memory. Hippocampal learning and memory deficits were evaluated using the Morris water maze paradigm as previously described[29], with additional modifications. Specifically, the water maze apparatus consisted of a 4ft diameter pool filled with opaque water (25°C; colored with non-toxic Crayola® white paint). Visual cues were placed on the walls surrounding the pool and a platform (4 inches in diameter) was hidden below the surface of the water (1 cm). Initial training consisted of four trials per day over four days. Mice were introduced into the pool at variable entry points, with every entry point used over the course of the day. The location of the platform remained constant throughout training period. The mice were given 60 sec to locate platform. On the fifth day of testing, a probe trial was conducted in which the platform was removed over one 60-second trial. Tracking software (Noldus Ethovision XT) was used to record swim speed, total distance traveled, distance traveled in platform quadrant, time spent in platform quadrant, and platform crossings.
Repetitive behaviors. To test mice for repetitive behaviors we used marble burying. For marble burying, we quantified the number of marbles a mouse buried over a 30-min period in a cage that contained extra bedding at a depth of 5 cm and 28 marbles arranged in a 4 × 7 grid[31]. Marbles were scored as buried if at least 60% covered.

Long-term potentiation (LTP). All electrophysiology experiments were performed on transverse hippocampal slices (400 μm) from 4 to 6-month-old mice. Slice preparation, and ACSF composition were performed as previously described[32]. LTP was induced with three trains of 1 s, 100 Hz high frequency stimulation (HFS) with an intertrain interval of 60 s. Field excitatory postsynaptic potentials (fEPSPs) were collected for an additional 140 min. Bipolar stimulating electrodes were placed at the border of area CA3 and area CA1 along the Schaffer-Collateral pathway. ACSF-filled glass recording electrodes (1–3 MΩ) were placed in stratum radiatum of area CA1. fEPSPs were amplified (A-M Systems Model 1800) and digitized (Digidata 1440; Molecular Devices) prior to analysis (pClamp; Molecular Devices). The initial slopes of the fEPSPs from averaged traces were normalized to those recorded during baseline.

Immunocytochemistry. Astrocytes were plated on uncoated sterile coverslips at 5×10^4 cells per well of a 24-well plate. For co-cultures, 7.5×10^4 neurons were mixed with 2.5×10^4 astrocytes and plated onto poly-D-lysine coated sterile coverslips. Cells were fixed in 4% PFA for 10 min immediately followed by permeabilization with 0.5% Triton X for 10 min and blocked with 10% Goat Serum. Primary antibodies were incubated overnight at 4°C followed by a 2 h incubation with secondary antibodies at RT. To visualize the nucleus of individual cells, Hoescht was applied in the final wash prior to mounting. *Free floating immunohistochemistry*. Cryosectioned free floating sagittal brain sections (30-40 μm thickness) of perfused and fixed mouse brains were collected in PBS and permeabilized in 0.5% Triton X for 10 min and blocked with 10% Goat Serum. Primary antibodies were incubated overnight at 4°C with slight rocking. The following day, secondary antibodies were added for 2 h at RT. To visualize the nucleus of individual cells, Hoescht was applied in the final wash prior to mounting. In addition to these steps, amplification kits were used for huGFAP immunostaining only. *Quantitative immunostaining*. ImageJ was used to semi automate the quantification of the colocalization of GFP with ALDH1L1 and GFAP in cryosectioned free floating sagittal brain sections. Composite images of the somatosensory cortex and hippocampus were acquired on epifluorescent microscope (Leica). To process, composite images were split into individual channels and transformed into 8 bit images. Standardized thresholding and processing parameters were applied to all images and then cells were manually counted with the help of the ROI manager tool.

Two-photon *in vitro* Ca^{2+} imaging. To acquire Ca^{2+} signals, astrocytes were loaded with a Ca^{2+} indicator dye (Fluo-4-am, 1μM) and incubated for 30 min at 37°C. The dye was completely washed away and cells permitted to equilibrate at 37°C for 30 min. We used Olympus RS equipped with a scanning galvanometer and a Spectra-Physics Mai Tai DeepSee laser for the excitation of GFP. We recorded fluorescence through gallium arsenide phosphide (GaAsP) detectors using the Fluoview acquisition software (Olympus) with a green light emission bandpass filter (Semrock). We acquired a time-lapsed image (512 x 512 pixels, 300 frames, 1 Hz.) for each coverslip per ASD or CTRL line. In addition to measuring spontaneous waves, we also measured stimulus evoked Ca^{2+} activity in CTRL or ASD astrocytes with ATP (50μM). To do this, we recorded spontaneous fluorescent activity for 2 min while buffer continuously flowed through the recording chamber. Perfusion was halted and ATP was manually added to the perfusion chamber and an additional 2 min of imaging was recorded. After 2 min, perfusion of recording buffer was turned back on. Data were sorted and segmented with Suite two-photon software, an open source toolbox for MATLAB[33]. The change in fluorescence over baseline fluorescence ($\Delta F/F_0$) was used to assess peak amplitude Ca^{2+} spikes at baseline and after application of ATP.

Cranial window implant surgery, recovery, and habituation. Adult (P60+) mice with transplanted human astrocytes expressing GCaMP6f were anesthetized with isoflurane (4% for induction, 1–1.5% vol/vol for maintenance) and placed in a stereotaxic frame (Kopf), with body temperature kept at ~37 °C with a feedback-controlled heating pad (Harvard Apparatus). After removing the scalp and clearing the skull of connective tissues, a custom-made titanium head-bar was fixed onto the skull with cyanoacrylate adhesive (Krazy Glue) and covered with black dental cement (Ortho-Jet). A circular craniotomy (3-mm diameter) was then performed above the border of primary somatosensory and motor cortices on the right hemisphere (right S1/M1, centered at 2.0 mm lateral from bregma). The bone was removed without damaging the underlying dura. Then a glass cranial window consisting of a 3-mm diameter #1 coverslip (150 μm thickness, Warner Instruments) was place on top of the dura, flush with the skull surface, and sealed in place using tissue adhesive (Vetbond, 3M). The exposed skull surrounding the cranial window was then completely covered with cyanoacrylate adhesive and then with black dental cement to build a small chamber, intended to hold liquid for imaging with water-immersion objective. Extreme care was taken to ensure that the dura experienced no damage or major bleeding before and after cranial window implantation. Mice with damaged dura or unclear window were discarded and not used for imaging experiments. After surgery, animals were returned to their home cages for at least 3 weeks for recovery, to provide ample time for astrocytes to return to their nonreactive state. After one week in recovery, animals were subjected to periodic handling and head-fixation for habituation to the imaging conditions. Animals were housed in reversed light-dark cycle (light on from 7pm-7am), and all habituation, imaging, and behavioral experiments were conducted during the dark/active phase for the animals.

Two-photon *in vivo* Ca^{2+} imaging. Astrocytes isolated/expanded from CTRL or ASD iPSC derived organoids were infected with AAV virus carrying the genetically encoded calcium indicator GCaMP6f (AAV2/5-*GfaABC$_1$D-GCaMP6f*). The GCaMP6f-expressing human astrocytes were engrafted into neonatal *Rag2*^{KO} by injecting the cells into the frontal cortex. We raised the transplanted mice to adulthood (P60+) before subjecting them to *in vivo* experiments. We implanted a 3-mm-diameter glass cranial window over the primary somatosensory and motor cortices (S1/M1), attached a titanium head-bar to the skull for head-fixation, and secured the implants with (black) dental cement. We allowed the mice to recover post-surgery for at least 3 weeks to provide ample time for astrocytes to return to their non-reactive state. During this time, we periodically subjected the mice to handling and head-fixation to habituate them to experimental conditions. Finally, we performed *in vivo* two-photon imaging to record Ca^{2+} activity in transplanted human astrocytes within live mice actively responding to the environment. Two-photon calcium imaging was performed with an Olympus multiphoton laser-

scanning microscope (FVMPE-RS) with a Ti-Sapphire laser (Spectra-Physics Mai Tai DeepSee) tuned to 920 nm, through a 25X, 1.0 NA water-immersion objective (Olympus XLPLN25XSVMP2). Photograph in **Fig. 3**a shows labeled components of our custom-designed floating platform, developed to provide a tactile virtual-reality environment for head-fixed mice and to enable imaging of actively locomoting animals with minimal motion confounds. The photo shows a mouse situated at the center under a microscope objective and on top of a platform comprised of a 15x15 cm plastic container with 1cm depth, enclosed by a thin sheet of flexible plastic film material as walls, and filled with bedding material from the animal's home cage. The platform floats on water inside a larger plastic container (40 x 40 x 8 cm). The animal's head is immobilized by fixation of its attached head-bar to a custom-built holder consisting of a heavy stable base assembled with goniometers and multi-axis micro-positioning devices for making fine adjustments to the position and angle of the animal's head. While head-fixed, the animal's body movement on the floating platform directly translates into platform movement, akin to moving on a treadmill or tactile virtual-reality. The fluidity of water allows the forces generated by the animal movement on the platform to be absorbed by the water instead of causing vibration of the head relative to the objective lens and resulting in image motion artifacts. The imaging setup includes an air puff delivery tube (3mm inner-diameter PVC tubing) connected to an air compressor on one end and positioned at 1cm away from the animal's face/nostril on the other for delivering the startle stimulus. The setup also includes an infrared-sensitive camera and an infrared light for monitoring animal behavior and locomotion in the dark during 2-photon imaging. *Imaging setup (**Fig. 3**c):* (Left upper panel): a representative image of GCaMP6f-expressing human astrocytes in the cortex of a mouse engrafted with cells derived from a CTRL human subject. *Right, upper panel (orange box)*: Ca^{2+} transient recorded from the cell marked by the orange circle on the left image, showing an increase in Ca^{2+} level after the startle/air-puff stimulus (*orange arrow*). Ca^{2+} level was sustained at an elevated level (*gray line/arrow* = +5.5% ΔF/F) for the duration of the recording. Note the images were acquired at 15Hz (15 frames per second) using one-way resonant scanning, at a much higher rate than typical astrocyte two-photon imaging using galvanometer-based scanning (less than 1Hz). This higher acquisition rate has enabled us to record Ca^{2+} fluctuation dynamics in much higher temporal resolution, in addition to allowing for more robust image motion correction by minimizing in-frame motion artifacts. (Left bottom panel): The objective is placed at the end of an articulating arm (representing the Olympus inner-focus articulating nosepiece); it is angled (15 degrees to the vertical) to allow the animal to assume a more natural head position, for ease of locomotion and stress minimization. The video camera icon on the left represents the infrared-sensitive camera recording the animal's behavior and locomotion (platform position). Videos were acquired at 15Hz to produce one-to-one corresponding frames between the two-photon images and video recordings. (Right bottom panel-gray box): Representative trace of

recorded locomotion during imaging, produced by analyzing the infrared video recording and plotting the light intensity change of a select ROI (region of interest) on the floating platform. As animal movement directly translated into platform displacement, any body motion generated by the animal could be tracked with accuracy and high temporal resolution, even heavy breathing could be seen indicated by the brief ticks at the latter part of the recording.

Data processing and analysis of *in vivo* Ca^{2+} imaging. For the present study, extra care was taken to minimize potential motion confounds, with extensive habituation to reduce animals' stress and the implementation of a newly designed floating platform that allowed the force of the animal's movements to be absorbed by the fluidity and buoyancy of the water supporting the platform, especially in the up-down direction to mitigate the hard-to-correct z-axis motions. Resonant imaging at 15Hz also contributed to avoiding in-frame motion confounds and allowing minor between-frame shifts to be effectively corrected by accurate rigid-body transformations using the TurboReg plugin for ImageJ. Motion-registered data were then processed and analyzed following the steps outlined in Srinivasan and Huang *et al.*[48] An ROI-based approach is used to segment astrocytes in a semi-automated manner based on maximum-intensity projections of background-subtracted image stacks. As compared to previous studies with mouse astrocytes, the transplanted human astrocytes displayed high basal fluorescence levels and tend to be clustered instead of being spread out in typical non-overlapping manner. These factors, combined with the feature of human astrocytes possessing fine processes that are radially distributed around the soma, made it difficult to resolve the fluorescence from processes vs. the soma to distinguish these different compartments. The ROI selected were assumed to encompass both soma and microdomains in processes. The post-startle percent changes in calcium levels were calculated by subtracting the mean fluorescence value from a pre-startle stable period (first 100 timepoints, 6.6 s) from the mean value form a post-startle stable period (last 100 timepoints, 6.6 s), divided by the initial mean F. The cells showing percent increase above +2% were categorized into the Increased Response group, those below -2% into the Decreased Response group, and those in between +2 and -2% in the Unchanged group. The percent change in variance were calculated similarly as above but using the standard deviations as the measure. Statistical comparisons were made using unpaired two-tailed Student's t test.

Astrocyte/neuron co-cultures. For the preparation of primary neurons, cells were dissociated from the brains of E18 C57/6N embryonic mouse pups. After the removal of adherent meninges, hippocampi were removed and dissociated into a single cell suspension with trituration following incubation for 15 min with 0.1% TRYP-LE as previously outlined[34]. Cells were counted with a hemocytometer and a mixture of

neurons was added to a mixture containing astrocytes in a 4:1 (dissociated hippocampal cells:human astrocytes) ratio. This mixed cell suspension was plated onto 24x50mm, No. 1.5 sterilized glass coverslips or 48-well multielectrode array culture plates that were precoated with 0.01% poly-D-lysine. Cultures were maintained for the first 24 h in 50% neural basal media supplemented with B27 and 50% astrocyte base media. After the initial plating, subsequent feeds contained 100% neural basal media supplemented with B27, 1% glutamine, and penicillin/streptomycin antibiotic. Co-cultures were housed at 37°C in a humidified 5% CO_2 containing atmosphere.

Neuronal network activity. Recordings on multielectrode arrays (MEAs) began at DIV 14. The MEA system (Maestro system from Axion BioSystems) provided a non-invasive extracellular recording method that did not damage neuronal viability *in vitro.* Using the 48-well multielectrode array (MEA) plates from Axion Biosystems, we plated primary mouse neurons with astrocytes derived from 4 ASD lines and 3 control lines in triplicate. A non co-culture control was added for each MEA recording. This included neurons dissociated from the same litter but without any human astrocytes. These non-human control cultures were subjected to the same conditions as mouse neuron/human astrocyte co-cultures. Each well was precoated with 0.01% poly-D-lysine and seeded with $7.5x10^5$ neurons and $2.5x10^5$ astrocytes. Co-cultures were fed twice a week and measurements were taken at DIV14. Recordings were performed with the Maestro Pro MEA system and spike sorting and analyses performed in AxIS software from Axion Biosystems. Each well contained 16 electrodes (arranged in 4x4 grid) that detected changes in extracellular field potentials reflecting the spiking activity of neurons. Electrodes with an average of ≥5 spikes/min were defined as active. Spikes were detected when they were ≥5.5x the standard deviation of the raw signal per electrode. Each plate equilibrated for a minimum of 10 min prior to recording in Maestro Pro Instrument. Recordings occurred within a chamber heated to 37°C with 5% CO_2.

Spine density. Primary hippocampal neurons were dissociated from the hippocampi of C57BL/6N embryos at E17-18 as previously described with some modifications[34] and plated $1x10^5$ neurons with $4x10^4$ astrocytes isolated from organodis derived from CTRL or ASD iPSCs on 0.01% poly-D-lysine coated 12 mm round glass coverslips. To visualize dendrites and dendritic spines, neurons were infected with an adeno-associated virus that expressed GFP under the control of the α*CaMKII* promoter. This promoter-specific expression of GFP restricted labeling to only excitatory neurons and their spines. To perform spine analysis, we fixed neurons at DIV18 with 4% PFA, amplified the GFP signal with antibodies and fluorescent immunochemistry. We acquired images by generating maximum intensity projections from z-stacks using optical sections (Olympus confocal microscopy, 0.35 μm per section). To quantify, we

identified a 10 μm dendritic segment at least 20 μm away from the soma and counted individual spines using ImageJ.

Knockdown of IP_3Rs and high throughput Ca^{2+} detection assay. To modulate Ca^{2+} transients in ASD astrocytes, lentiviruses carrying shRNAs against IP_3R 1 and 2 (SMARTvector Lentiviral Human ITPR1 hCMV-TurboRFP shRNA and SMARTvector Lentiviral Human ITPR2 hCMV-TurboRFP shRNA; purchased from Dharmacon, see Key Resources table for further information) were used. At passage 8, ASD astrocytes were plated in 6-well plates at low densities. After 24 hours, media was changed to 500 μL of serum free media including 5 μL of targeting or control nontargeting (see Key Resources table) lentiviruses. 6 h later media was supplemented with 1 mL of astrocyte expansion media with 2% serum. The cells were either used for transplantation or high throughput Ca^2 detection assay. Before performing these experiments, downregulation of both receptors in ASD astrocytes was validated by western blotting (**Supp Fig. 10**) (see Key Resources table for antibody information). Unlike genetic ablation such as knockout, viral knockdown of a gene is typically incomplete. Thus, it is expected that our strategy would modulate but not eliminate Ca^{2+} releases. Given that ASD astrocytes displayed increased Ca^{2+} transients compared to control astrocytes, partial knockdown could normalize Ca^{2+} transients closer to control levels. For high throughput Ca^{2+} detection assay, astrocytes were seeded on clear bottom black 96-well plates at $1x10^4$ astrocytes per well 24 h prior to recording. The following day, cells were loaded with Ca^2 indicator dye optimized for use in high throughput systems (1 μM Fluo-8-AM). Astrocytes were incubated with Fluo-8-AM for 30 min at 37°C. The plate was placed in a preheated plate reader (Hammatsu FDSS 6000) and recording occurred in Ca^2 free HBSS at 37°C. Baseline fluorescence was recorded for 5 min (470–495 nm excitation and 515–575 nm emission). Next, a robotic arm applied G_s cocktail, forskolin, and thapsigargin to predetermined wells. The recipe and final concentration of G_q cocktail was as follows: 50 μM DHPG, 50 μM norepinephrine, 50 μM ATP, and 50 nM Endothelin diluted in HBSS. Forskolin (final concentration 12.5 nM) and thapsigargin (final concentration: 2 μm) were also diluted in HBSS. After application of stimulators, recording continued for 10 additional min. Recordings were performed in duplicate or triplicate. The peak change in fluorescence amplitude (ΔF) in each well was normalized to the basal fluorescence of that well before stimulation (F_0). All data are represented as mean of $\Delta F/F_0$ from each corresponding cell line from the same plate.

Glutamate Assay. Astrocytes were grown in T-25 flasks with a starting density of $3x10^5$. Supernatants were collected at 96 hours (no media change). Astrocytes were dissociated and counted after media collection for normalization. Glutamate assay was performed according to kit instructions (Abcam, ab83389) and read in a 450-nm microplate reader.

Neural progenitor expansion. iPSC colonies were enzymatically dissociated with 1X Accutase® solution in dulbecco′s phosphate-buffered saline (DPBS) without Ca^{2+} or Mg^2 at 37°C for 10 min. Next, cells were transferred to a 15-mL falcon tube, diluted in E8 media to inactivate the enzymatic reaction, and centrifuged @1000 RPM for all lines. iPSCs were resuspended in E8 media plus ROCK inhibitor and plated in 6-well plates that were precoated with 1:40 matrigel at a density of $1.5x10^6$ cells per well. Once wells reached ~70% confluency, media was changed from E8 to E6 to help promote the formation of a uniform neuroepithelial layer. In adherence to the dual SMAD inhibition protocol[4], E6 media was supplemented with SB-431542 and LDN 193189 factors. Media changes occurred daily and cells were observed for presence of a neuroepithelial sheet, in which cell bodies became smaller and more densely packed. This typically occurred in our hands between 5-8 days in E6+factor media. Timing of neuroepithelial development varied slightly between each independent line. Between days 5-8, progenitors were enzymatically dissociated with Accutase® solution, prepared as a single cell suspension in DPBS, and housed on ice prior to transplantation into the brains of $Rag2^{KO}$ newborn pups between postnatal day 1 and 3 (P1-3).

Data analysis. Data was analyzed as described in figure legends. Typically, this involved parametric hypothesis testing via *t* tests and ANOVA. Data were corrected for all relevant comparisons, as outlined in-text, and typically comprised Bonferroni or Tukey's adjustment. Data were typically represented as mean ± SEM. Relevant variations to analysis (e.g., calcium imaging and proteomics) are described in the relevant section of methods.

Acknowledgements
We thank the WCM Proteomics Core facility for providing experimental consultation and results. Especially, Dr. Guoan Zhang, the director, and two associate members, Mengmeng Zhu and Taojunfeng Su. We also thank the WCM Genomics Resources Core Facility for consultation and WES sequencing. Additionally, we thank Dr. Zhengming Chen, Ph.D., M.P.H., M.S., a senior research biostatistician in the Division of Biostatistics and Epidemiology at Weill Cornell Medicine, for statistical power analysis. We would like to thank Dr. Lavo Ramos-Espiritu and the High Throughput and Spectroscopy Resource Center at Rockefeller University for training on plate reader for Ca^2 imaging experiments and valuable comments. This work was supported by a NIH grant 1R01MH120156-01 to D.C.

Author contributions
D.C. and M.A. conceived of the project, designed most of the experiments and wrote the manuscript with input from all authors. B.S.H. designed and performed live-animal Ca^{2+} imaging experiments, analyzed the data, and helped with the manuscript. P.W. analyzed the WES data and prepared the Supplementary Tables 2 and 3 with input

from M.E.R. M.J.N. assisted with 3D cerebral organoid cultures and performed all fear-conditioning and marble burying tests with the help of E.B.A. and A.L. M.A. performed co-culture and other behavioral experiments. M.A., A.L. and E.B.A. executed iPSC and murine cell culture experiments, immunohistochemical experiments, and associated analyses. P.J.L. performed re-analysis for some of the behavioral data. M.A. and J.W. performed *in vitro* Ca^{2+} imaging experiments with input from C.L. F.L. performed and analyzed LTP experiments with input from E.K. D.G. and M.C. performed proteomics analysis.

Supplementary References

1. Barbar, L., *et al.* CD49f Is a Novel Marker of Functional and Reactive Human iPSC-Derived Astrocytes. *Neuron* **107**, 436-+ (2020).
2. Zamanian, J.L., *et al.* Genomic Analysis of Reactive Astrogliosis. *Journal of Neuroscience* **32**, 6391-6410 (2012).
3. Beaudoin, G.M.J., *et al.* Culturing pyramidal neurons from the early postnatal mouse hippocampus and cortex. *Nat Protoc* **7**, 1741-1754 (2012).
4. Chambers, S.M., *et al.* Highly efficient neural conversion of human ES and iPS cells by dual inhibition of SMAD signaling. *Nat Biotechnol* **27**, 275-280 (2009).
5. Sauvageot, C.M. & Stiles, C.D. Molecular mechanisms controlling cortical gliogenesis. *Curr Opin Neurobiol* **12**, 244-249 (2002).
6. Mariani, J., *et al.* FOXG1-Dependent Dysregulation of GABA/Glutamate Neuron Differentiation in Autism Spectrum Disorders. *Cell* **162**, 375-390 (2015).
7. McCarroll, S.A. & Hyman, S.E. Progress in the genetics of polygenic brain disorders: significant new challenges for neurobiology. *Neuron* **80**, 578-587 (2013).
8. Losh, M., *et al.* Neuropsychological profile of autism and the broad autism phenotype. *Arch Gen Psychiatry* **66**, 518-526 (2009).
9. Constantino, J.N. The quantitative nature of autistic social impairment. *Pediatr Res* **69**, 55R-62R (2011).
10. Gamliel, I., Yirmiya, N., Jaffe, D.H., Manor, O. & Sigman, M. Developmental trajectories in siblings of children with autism: cognition and language from 4 months to 7 years. *J Autism Dev Disord* **39**, 1131-1144 (2009).
11. Pickles, A., *et al.* Variable expression of the autism broader phenotype: findings from extended pedigrees. *J Child Psychol Psychiatry* **41**, 491-502 (2000).
12. Warren, Z.E., *et al.* Neurocognitive and behavioral outcomes of younger siblings of children with autism spectrum disorder at age five. *J Autism Dev Disord* **42**, 409-418 (2012).
13. Ben-Yizhak, N., *et al.* Pragmatic language and school related linguistic abilities in siblings of children with autism. *J Autism Dev Disord* **41**, 750-760 (2011).
14. Gamliel, I., Yirmiya, N. & Sigman, M. The development of young siblings of children with autism from 4 to 54 months. *J Autism Dev Disord* **37**, 171-183 (2007).

15. Constantino, J.N., *et al.* Autistic social impairment in the siblings of children with pervasive developmental disorders. *Am J Psychiatry* **163**, 294-296 (2006).
16. Constantino, J.N. & Todd, R.D. Intergenerational transmission of subthreshold autistic traits in the general population. *Biol Psychiatry* **57**, 655-660 (2005).
17. Brennand, K.J., *et al.* Modelling schizophrenia using human induced pluripotent stem cells. *Nature* **473**, 221-225 (2011).
18. Marchetto, M.C., *et al.* Altered proliferation and networks in neural cells derived from idiopathic autistic individuals. *Mol Psychiatry* **22**, 820-835 (2017).
19. Soliman, M.A., Aboharb, F., Zeltner, N. & Studer, L. Pluripotent stem cells in neuropsychiatric disorders. *Mol Psychiatry* **22**, 1241-1249 (2017).
20. Stern, S., *et al.* Neurons derived from patients with bipolar disorder divide into intrinsically different sub-populations of neurons, predicting the patients' responsiveness to lithium. *Mol Psychiatry* **23**, 1453-1465 (2018).
21. Windrem, M.S., *et al.* Human iPSC Glial Mouse Chimeras Reveal Glial Contributions to Schizophrenia. *Cell Stem Cell* **21**, 195-208 e196 (2017).
22. Young-Pearse, T.L. & Morrow, E.M. Modeling developmental neuropsychiatric disorders with iPSC technology: challenges and opportunities. *Curr Opin Neurobiol* **36**, 66-73 (2016).
23. Hasel, P., *et al.* Author Correction: Neurons and neuronal activity control gene expression in astrocytes to regulate their development and metabolism. *Nat Commun* **9**, 16176 (2018).
24. Foo, L.C., *et al.* Development of a Method for the Purification and Culture of Rodent Astrocytes. *Neuron* **71**, 799-811 (2011).
25. Zamanian, J.L., *et al.* Genomic analysis of reactive astrogliosis. *J Neurosci* **32**, 6391-6410 (2012).
26. Han, X., *et al.* Forebrain engraftment by human glial progenitor cells enhances synaptic plasticity and learning in adult mice. *Cell Stem Cell* **12**, 342-353 (2013).
27. Windrem, M.S., *et al.* Neonatal chimerization with human glial progenitor cells can both remyelinate and rescue the otherwise lethally hypomyelinated shiverer mouse. *Cell Stem Cell* **2**, 553-565 (2008).
28. Zhao, C., Teng, E.M., Summers, R.G., Jr., Ming, G.L. & Gage, F.H. Distinct morphological stages of dentate granule neuron maturation in the adult mouse hippocampus. *J Neurosci* **26**, 3-11 (2006).
29. Allen, M., *et al.* Protease induced plasticity: matrix metalloproteinase-1 promotes neurostructural changes through activation of protease activated receptor 1. *Sci Rep* **6**, 35497 (2016).
30. Notaras, M., *et al.* UPF2 leads to degradation of dendritically targeted mRNAs to regulate synaptic plasticity and cognitive function. *Mol Psychiatry* (2019).
31. Santini, E., *et al.* Exaggerated translation causes synaptic and behavioural aberrations associated with autism. *Nature* **493**, 411-415 (2013).
32. Kaphzan, H., *et al.* Genetic reduction of the alpha1 subunit of Na/K-ATPase corrects multiple hippocampal phenotypes in Angelman syndrome. *Cell Rep* **4**, 405-412 (2013).
33. Moda-Sava, R.N., *et al.* Sustained rescue of prefrontal circuit dysfunction by antidepressant-induced spine formation. *Science* **364**(2019).
34. Beaudoin, G.M., 3rd*, et al.* Culturing pyramidal neurons from the early postnatal mouse hippocampus and cortex. *Nat Protoc* **7**, 1741-1754 (2012).
